# Crystal structure of the EcoKMcrA N-terminal domain (NEco): recognition of modified cytosine bases without flipping

**DOI:** 10.1093/nar/gkz1017

**Published:** 2019-11-14

**Authors:** Anton Slyvka, Evelina Zagorskaitė, Honorata Czapinska, Giedrius Sasnauskas, Matthias Bochtler

**Affiliations:** 1 International Institute of Molecular and Cell Biology, Trojdena 4, 02-109 Warsaw, Poland; 2 Institute of Biotechnology, Vilnius University, Saulėtekio av. 7, 10257 Vilnius, Lithuania; 3 Institute of Biochemistry and Biophysics PAS, Pawinskiego 5a, 02-106 Warsaw, Poland

## Abstract

EcoKMcrA from *Escherichia coli* restricts CpG methylated or hydroxymethylated DNA, and may act as a barrier against host DNA. The enzyme consists of a novel N-terminal specificity domain that we term NEco, and a C-terminal catalytic HNH domain. Here, we report that NEco and full-length EcoKMcrA specificities are consistent. NEco affinity to DNA increases more from hemi- to full-methylation than from non- to hemi-methylation, indicating cooperative binding of the methyl groups. We determined the crystal structures of NEco in complex with fully modified DNA containing three variants of the Y^5m^CGR EcoKMcrA target sequence: C^5m^CGG, T^5m^CGA and T^5hm^CGA. The structures explain the specificity for the two central base pairs and one of the flanking pairs. As predicted based on earlier biochemical experiments, NEco does not flip any DNA bases. The proximal and distal methyl groups are accommodated in separate pockets. Changes to either pocket reduce DNA binding by NEco and restriction by EcoKMcrA, confirming the relevance of the crystallographically observed binding mode in solution.

## INTRODUCTION

*Escherichia coli* McrA (EcoKMcrA) has attracted interest for over 30 years as one of the natural barriers against genetic engineering of *E. coli* K strains (1–3). The protein potently restricts methylated and hydroxymethylated DNA (from the glucosyltransferase deficient T4gt or T6gt phage), but not glucosyl-hydroxymethylated DNA (from wild-type T4 phage) (1,4,5). The restriction activity of EcoKMcrA on modified DNA is sequence context dependent. The specificity of the full-length enzyme for (Y)^5m^CGR target sequences has been inferred from a combination of restriction experiments in cells (1), and electrophoretic mobility shift assays (EMSAs) (6). While binding of EcoKMcrA to modified DNA can be readily confirmed in the test tube, the cleavage of DNA by the enzyme has been elusive.

Very recently, we demonstrated the weak *in vitro* nuclease activity of EcoKMcrA and determined the crystal structure of the enzyme (4). The structure confirmed the presence of the predicted HNH nuclease domain at the C-terminus (7). Moreover, it showed that the N-terminal domain of EcoKMcrA, termed NEco in this work, is distantly similar to I-DreI, a dimeric meganuclease composed of I-DmoI and I-CreI homing endonucleases (8) and to MotA, a transcription factor from T4 phage (9). NEco did not exhibit sequence or structural similarity to SRA domains previously implicated in binding of modified DNA bases by nucleotide flipping (10–12), and to zinc finger or MBD proteins, known to bind modified cytosine bases without flipping (13,14). In the absence of a co-crystal structure of EcoKMcrA with DNA, it was possible to propose an approximate DNA binding mode based on the surface charge distribution, sequence conservation and small-angle X-ray scattering (SAXS) data (4). However, it was not possible to deduce details of methyl- or hydroxymethyl recognition.

Here, we report crystal structures of the EcoKMcrA N-terminal domain (NEco, residues 1–143) in complex with fully modified methylated and hydroxymethylated DNA targets. The structures show that monomeric NEco has two separate pockets for (hydroxy)methyl groups on cytosines, reachable without nucleotide flipping, and reveal the mechanistic basis for the Y^5(h)m^CGR sequence specificity of EcoKMcrA. In order to demonstrate the biological relevance of the observed DNA binding mode, we designed variants of the enzyme differing in the key residues expected to play a role in specificity. The effect of the amino acid substitutions was tested in the context of the full-length protein by restriction assays and in the context of the N-terminal domain alone by an electrophoretic mobility shift assay. The results show that NEco recognizes cytosine-modified DNA differently than previously known modification specific domains.

## MATERIALS AND METHODS

### NEco expression and purification

Full-length EcoKMcrA was expressed from pLATE31 plasmid, NEco was expressed from pET15b-mod plasmid with an additional stop codon to terminate the protein after amino acid 143. Expression constructs for variants were generated from those for the wild-type by site directed mutagenesis (by PCR and DpnI digestion of dam^+^ PCR template).

For crystallography, NEco was expressed for 16 h at 25°C after induction with 0.25 mM IPTG. Cells were harvested and lysed in the presence of 1 mM PMSF. Supernatants were applied to a HisTrap column (GE Healthcare) in 50 mM Na-phosphate pH 8.0, 750 mM NaCl, 20% glycerol, 10 mM 2-mercaptoethanol (application in 1 mM imidazole, washes in 10 mM imidazole, and elution with the imidazole gradient). The eluate was diluted to bring the NaCl concentration down to 50 mM. It was then applied to a Heparin HP column (GE Healthcare) in 50 mM Na-phosphate pH 8.0, 50 mM NaCl, 10% glycerol, 10 mM 2-mercaptoethanol and eluted with 50–800 mM NaCl gradient. NEco containing fractions were dialyzed against the crystallization buffer (20 mM Tris–HCl, pH 8.0, 100 mM NaCl, 1 mM EDTA and 2 mM DTT). After the dialysis the protein was either used for crystallization or mixed 1:1 with glycerol and stored at −20°C.

For biochemistry, NEco and its variants (S30 was substituted with A, L or V, W31 with A, F, H, I, L, S, V, Y and N119 with A) were expressed overnight at 16°C after induction with 0.2 mM IPTG. Cells were harvested and lysed. Supernatants were subjected to a HisTrap column (GE Healthcare, 18 mM Tris–HCl pH 8.0, 450 mM NaCl and 10% glycerol, elution in imidazole gradient), a desalting column to reduce NaCl concentration, and then heparin chromatography (GE Healthcare, 10 mM Tris–HCl, pH 8.0, elution in 0.1–1 M NaCl gradient). The purified NEco was rebuffered into storage buffer (20 mM Tris–HCl pH 8.0, 200 mM KCl, 1 mM DTT, 50% v/v glycerol) and stored at −20°C. The structural integrity of the variants was confirmed by circular dichroism.

### Crystallization and structure determination

NEco in the crystallization buffer was concentrated to 8.7 mg/ml (0.5 μM) using Amicon Ultra-15 3 MWCO (Merck Millipore) and mixed in the 1:1.2 ratio with 10-bp oligoduplex of 5′-TCAXXXXTTC-3′/5′-GAAXXXXTGA-3′ sequence, where XXXX stood for either C^5m^CGG, T^5m^CGA or T^5hm^CGA. Final protein:DNA concentrations were 436:523 μM for the C^5m^CGG complex and 626:751 μM for the T^5m^CGA and T^5hm^CGA complexes. Diffracting crystals were grown by mixing 1.8 μl of the protein–DNA mixture with 2.2 μl of the condition F1 of the PACT premier crystal screen (Molecular Dimensions) (0.2 M NaF, 0.1 M Bis–Tris propane, pH 6.5, 20% PEG3350). Crystals were grown by the vapor diffusion method in hanging drops in Linbro plates. They were cryo-protected by the addition of 25% glycerol and flash-cooled in liquid nitrogen, and diffracted up to approximately 2 Å on synchrotron beamlines. The raw diffraction data were deposited at the REPOD database (http://dx.doi.org/10.18150/repod.1407597). The structure of the first NEco–DNA complex (containing the C^5m^CGG target) was solved by molecular replacement using the Phaser program (15) and the NEco domain from the previously determined structure (PDB code: 6GHC, residues 1–143) (4). The program readily found two molecules of the domain in the asymmetric unit of the crystal. The other NEco–DNA complexes were then solved using the originally determined structure as the starting model. The structures were automatically rebuilt using the ARP/wARP program (16). The two DNA molecules were added manually. The programs REFMAC (17), Phenix (18) and COOT (19) were used for refinement. The data collection and refinement statistics are presented in Supplementary Table S1. The final model coordinates and the corresponding structure factors were deposited in Protein Data Bank under accession codes: 6R64 (C^5m^CGG), 6T21 (T^5m^CGA) or 6T22 (T^5hm^CGA).

### DNA binding studies

DNA-binding of EcoKMcrA N-terminal domain was analyzed by the electrophoretic mobility shift assay (EMSA) using ^33^P or ^32^P-labeled oligoduplexes with selected target site variants (Supplementary Table S2). DNA concentration was 10 nM, protein concentrations varied from 5 to 2000 nM. Please note that the domain concentrations are used throughout this study. The full length protein is a dimer and contains two NEco domains per molecule. Samples were prepared and electrophoresed as described previously (4). All experiments with wt NEco were repeated at least three times. The samples in EMSA competition experiments contained 10 nM radiolabeled DNA, 10 nM NEco domain, and various concentrations (3–3000 nM) of unlabeled competitor DNA. Quantification of DNA binding and competition data was performed as described in Supplementary Methods and previously (20). The conventional gel-shift experiments (concentration fixed for DNA and variable for protein) performed with wt NEco and suboptimal DNA substrates, and with some NEco variants did not yield quantifiable protein–DNA complexes, thereby precluding direct assessment of DNA binding affinities. Moreover, our gel-shift experimental setup was not optimized for measuring *K*_D_s that are relatively small, i.e. comparable to the DNA concentrations used in our assays (10 nM). We therefore present these experiments (and, where possible, calculated *K*_D_s) as merely qualitative representation of DNA binding by wt NEco and its variants. For quantitative analysis, we have used an EMSA-based competition assay, which allowed accurate and sensitive comparison of binding affinities to optimal and suboptimal DNA sequences.

### EcoKMcrA toxicity test

BL21(DE3) (McrA^−^) *E. coli* cells with no intrinsic antibiotic resistance were transformed with 18 ng of pLATE31 plasmids (Amp^R^) containing an open reading frame for wild type EcoKMcrA, its H229A catalytic variant, or the variants containing single amino acid substitutions in the modified base binding pockets (S30 was mutated to A, L or V, W31 to A, F, H, I, L, S, V, Y and N119 to A). At the recovery step after the heat-shock, the cells were supplemented with 0.9 ml of LB medium containing 1% glucose. After 1 h of shaking, 0.1 ml of the transformed cells was spread on LB agar plates that contained Amp and 1% glucose. The colonies were counted after overnight incubation at 37°C.

### Plasmid restriction assay

The plasmid restriction assay was performed as previously described (4). Briefly, BL21(DE3) (McrA^–^) *E. coli* cells with no intrinsic antibiotic resistance were transformed with pLATE31 plasmids (Amp^R^) containing wt EcoKMcrA or the variants listed above. The cells were made competent under Amp selection (at all growing steps the media contained 1% glucose). The cells were next transformed with pACYC184 plasmids (Cm^R^) containing either no methyltransferase (‘empty’) or M.HpaII gene. The pACYC184 plasmids were amplified in ER2267 (McrA^−^) *E. coli* strain and verified by sequencing. 30 ng (3 μl) was used for transformation of 0.1 ml of competent cells. At the recovery step after heat-shock the cells were supplemented with 0.9 ml of LB medium containing 1% glucose. After 1 h of shaking, cells were gently sedimented and resuspended in 0.6 ml of glucose-free medium. 0.1 ml of transformants was spread on LB agar plates that contained Amp, Cm and 1% glucose. The colonies were counted after overnight incubation at 37°C.

## RESULTS

### NEco expression and co-crystallization with DNA

Prior biochemical data on EcoKMcrA show that its N-terminal domain binds DNA in a methylation dependent manner (4). In our previous work, we used a longer fragment containing the N-terminal domain (residues 1–174), designed before the crystal structure of the full-length EcoKMcrA was solved and the accurate domain boundaries were known (4). As we expected that the extra C-terminal fragment may interfere with crystallization, we engineered a new expression construct ending at the precise domain boundary (NEco, residues 1–143) with the N-terminal MGHHHHHHEF tag.

The purified NEco was co-crystallized with 10mer dsDNA with the central C^5m^CGG, T^5m^CGA and T^5hm^CGA sequences. All three duplexes match the EcoKMcrA Y^5(h)m^CGR consensus. Crystals were grown in the hexagonal P6(1)22 space group and diffracted to 2.07–2.64 Å resolution. They contained two molecules of NEco, each bound to a duplex DNA. The structures were solved by molecular replacement and completed by manual building (Supplementary Table S1).

### NEco structure and DNA binding mode

**Figure 1.**
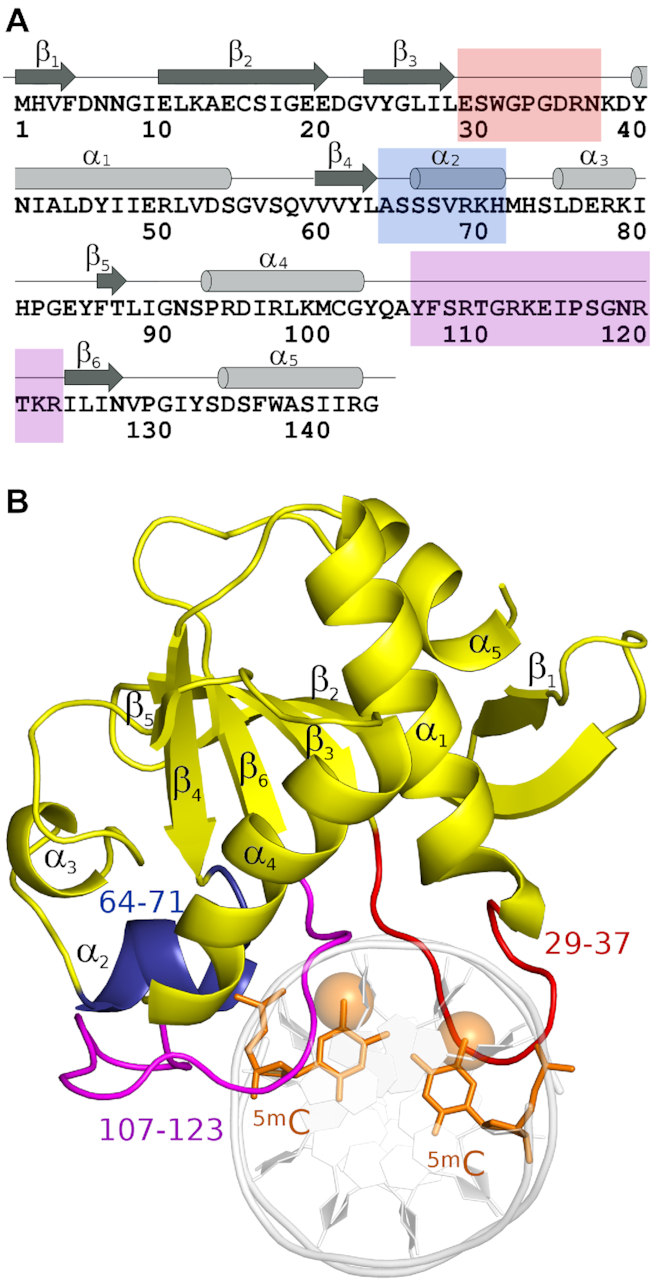
Overall structure of the EcoKMcrA N-terminal domain (NEco)–DNA complex. (**A**) Secondary structure diagram indicating three segments involved in sequence-specific DNA binding. (**B**) Ribbon representation of the NEco–C^5m^CGG DNA complex structure. The secondary structure elements are labeled. The methyl groups of the ^5m^C bases are shown as transparent spheres. The overall protein and DNA conformations are very similar in all three NEco–DNA structures presented here (Supplementary Figure S1).

**Figure 2.**
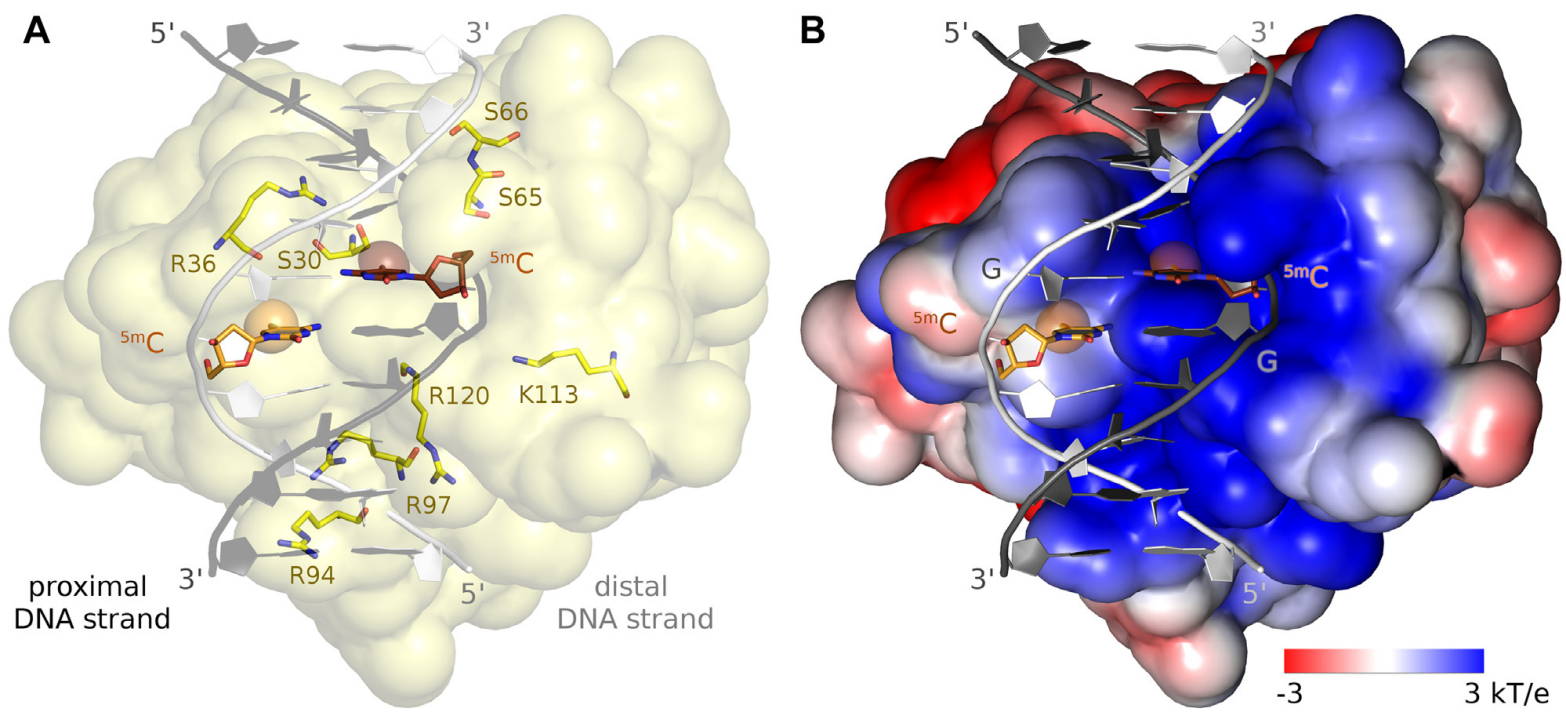
NEco-DNA binding mode. (**A**) The overview of the DNA binding groove in NEco with the phosphodiester backbone binding residues indicated. (**B**) The charge distribution on the surface of NEco. The proximal DNA strand that binds in the positively charged groove of the domain is indicated in dark gray color, the distal DNA strand is shown in white. The charge distribution was calculated with the DelPhi server (36).

The protein-DNA complexes in the three NEco structures are highly similar (all atom rmsd between 0.3 and 0.9 Å, Supplementary Figure S1, Supplementary Table S3). The conformation of DNA-bound NEco is also very similar to the one previously seen in the structure of the full-length protein dimer in the absence of DNA (4) (all atom rmsd between 1.2 and 1.6 Å, Supplementary Figure S2, Supplementary Table S3). Apparently, the protein can be bound with very little adaptive fit to the DNA. The NEco fold has already previously been described as organized around a six-stranded, anti-parallel β-sheet (4) (Figure 1). The domain has a predicted isoelectric point near neutral pH, which results from a balance of positively charged residues clustered on one face, and negatively charged residues on the other. The co-crystal structure of NEco confirms that the DNA is bound on the positively charged side of the domain (Figure 2). Although there are considerable distortions compared to the ‘canonical’ B-DNA form, Watson–Crick pairs are not disrupted. The absence of base flipping is consistent with the previously reported finding that EcoKMcrA does not increase pyrrolocytosine fluorescence, even when the analogue is present in the context of its target sequence (4).

The phosphodiester backbones of the two DNA strands engage in salt bridges and hydrogen bonds with NEco. The backbone of one strand, henceforth termed the ‘proximal’ strand, interacts with the protein in the region of the ^5(h)m^CpG step. Contacts include several salt bridges (with side chains of K113 and R36) and hydrogen bonds (with side chains of S30, S65 and S66). The backbone of the other DNA strand makes no contacts with NEco in the region of the central two target base pairs. However, it interacts with the protein about half a turn upstream (five 2′-deoxynucleotides), by several salt bridges (with side chains of R94, R97 and R120). In the following, we refer to this DNA strand as ‘distal’ (Figure 2).

### Structural basis of NEco sequence specificity

**Figure 3.**
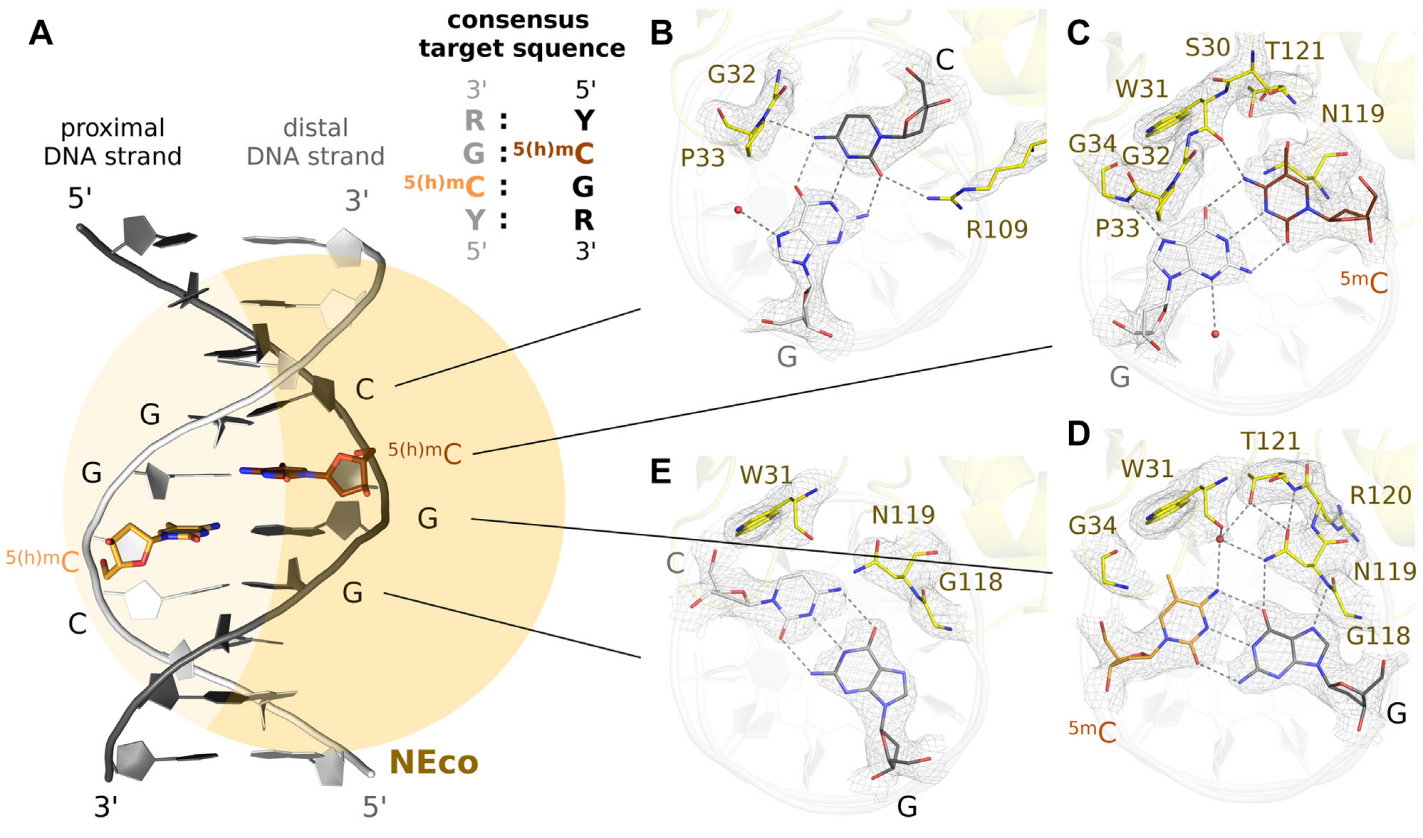
Structural basis of the NEco sequence and modification specificity. (**A**) Schematic view of the NEco-DNA complex and sequence context of the ^5(h)m^C residues preferred by NEco. (**B–E**) Interactions of the protein with the target sequence presented clockwise in the 5′ to 3′ direction of the proximal DNA strand. The proximal DNA strand is shown in dark gray, the distal strand in white and the protein is displayed in yellow. The composite omit map was contoured at 1.5 rmsd. The complex with C^5m^CGG target sequence was used as representative, but the interactions are largely preserved in all three structures (Supplementary Figures S1 and S3).

The EcoKMcrA N-terminal domain (NEco) engages in base-specific interactions with the DNA primarily via two loops (residues 29–37 and 107–123), as well as an α-helical region (residues 64–71) (Figure 1). All three regions of NEco approach the DNA predominantly from the major groove side. The loop 29–37 is closer to the distal strand, but nevertheless interacts with both DNA strands. The other two regions are closer to the proximal strand and interact only with it. NEco-DNA interactions are discussed below following the proximal strand in the 5′ to 3′ direction. The relatively scarce distal strand contacts are discussed together with interactions to the paired proximal strand base (Figure 3 and Supplementary Figure S3).

The proximal strand Y upstream of the ^5(h)m^CpG step accepts a hydrogen bond from R109 to its O2 atom. This contact is not sequence selective, because any base has a hydrogen bond acceptor in this outer minor groove position. When Y is a cytosine, the N4 atom of the C is about 3.3 Å away from peptide bond between G32 and P33. This distance would be suitable for an NH–π hydrogen bond (Figure 3B). When Y is thymine, there is altogether no possibility for hydrogen bond formation, but the extra 5-methyl group makes additional van der Waals contacts with the main chain carboxyl of G32 (Supplementary Figure S3B). How EcoKMcrA selects Y over R in this position is not clear. There is no obvious shape selection for a pyrimidine in the proximal or purine in the distal strand. However, the replacement of the Y:R base pair with R:Y pair would result in a mild clash of the purine with the opposite strand guanine from the ^5(h)m^C:G base pair (about 0.5 Å smaller interatomic distance than the sum of the van der Waals radii).

**Figure 4.**
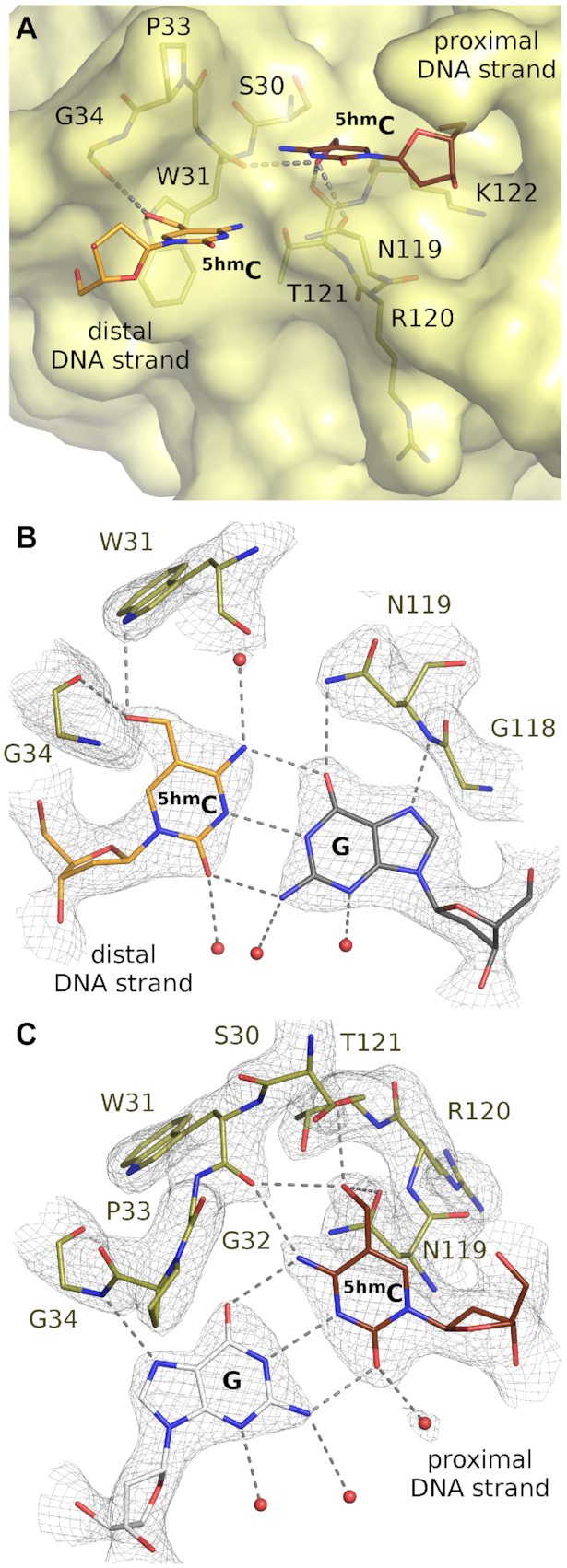
NEco binding pockets for the (hydroxy)methyl groups. (**A**) Surface representation of the ^5(h)m^C binding pockets. (**B**, **C**) Interactions of the proximal and distal strand hydroxymethyl groups. The three possible hydrogen bonds of the hydroxyl group of the proximal DNA strand ^5hm^C residue are indicated since it is difficult to unambiguously state which of them is formed. The composite omit map was contoured at 1.5 rmsd.

The proximal strand ^5(h)m^C and the paired distal strand G interact specifically with the main chain of the 29–37 loop. The ^5(h)m^C N4 atom donates a hydrogen bond to the main chain carbonyl of W31, and the guanine N7 atom accepts a hydrogen bond from the NH of G34. Together, these interactions suffice for non-degenerate recognition in this position (Figures 3C and 4).

The proximal strand G is specifically recognized by a hydrogen bond from the side chain carboxamide of N119 to the O6 atom of the base. The interaction is expected to be selective, because N119 also accepts a hydrogen bond from the main chain NH of T121, so that a flip of the carboxamide and reversal of the specificity (selection for A rather than G) is prevented. There are no direct interactions with the ^5(h)m^C base in the other strand. However, a water molecule is present in all three complexes (in both molecules of the asymmetric units), held by hydrogen bonds with the side chains of N119, T121, and the main chain carbonyl oxygen atom of W31. This water molecule is ideally positioned for accepting a hydrogen bond from the distal strand ^5(h)m^C base (Figures 3D and 4).

The interactions of the proximal strand R downstream of the ^5(h)m^CpG step and its paired Y base depend on whether the R:Y pair is a guanine–cytosine or an adenine-thymine pair. The methyl group of the distal strand T packs against the indole ring of W31. In all complexes, the Hoogsteen edge of the R base comes close to the main chain of G118. A pyrimidine base in this position would clash with this residue (considerably for C, very severely for T, since the 5-methyl group adds the steric bulk in the outer major groove region). Therefore we suspect that the enzyme uses shape selection to favor a purine (R) in this position (Figure 3E and Supplementary Figure S3NOP).

### Structural basis of NEco modification specificity

The binding pocket for the (hydroxy)methyl group in the proximal strand is a locally hydrophobic region, formed by the Cβ atom of S30 and a part of K122 (atoms Cα, Cβ and Cγ, Figure 4A). On the side, the methyl(ene) group is stacked against the side chain carboxamide of N119. The interaction with the asparagine may be responsible for the nearly absent propeller twist of the base pair, which contrasts starkly with the typical propeller twist of adjacent base pairs (Supplementary Table S4). In the crystal structure of NEco with ^5hm^C containing DNA, the hydroxyl group is oriented so that it comes within hydrogen bonding distance of the main chain carbonyl oxygen atoms of T121 and W31 and the Oδ atom of N119. In the absence of the information about the hydrogen atom positions it is impossible to unambiguously decide which of the three atoms is the actual hydrogen bond acceptor. The donor–acceptor distance criterion would point to N119 Oδ (2.7 Å versus 3.0 and 3.15 Å for the two carbonyl oxygen atoms) (Figure 4B).

The binding pocket for the (hydroxy)methyl group in the distal strand is primarily formed by the indole ring of W31 (Figure 4A). A hydroxymethyl group is also tolerated in this position, and donates a hydrogen bond to the main chain carbonyl of G34 (Figure 4C). In summary, despite the large accumulation of positive charges on the DNA binding surface of NEco, there are local hydrophobic patches that can accommodate the methyl(ene) parts of (hydroxy)methyl cytosines.

### Biochemical characterization of the NEco sequence specificity

The crystal structure suggests that NEco selects at least for the 5′-^5m^CGR-3′ in the proximal strand, and by implication, 5′-Y^5m^CG-3′ in the distal strand. Thus, the domain should bind Y^5m^CGR and reject R^5m^CGY target sequences (the complementary symmetric sequences are not shown for clarity). In case of the relaxed specificity for the first base pair, NEco should also bind to Y^5m^CGY/R^5m^CGR sites. The affinity of NEco to the DNA containing three types of targets was compared. As the domain binds best to fully methylated DNA (4), oligoduplexes containing fully methylated C^5m^CGG, G^5m^CGG/C^5m^CGC and G^5m^CGC were chosen as representatives of the three groups.

**Figure 5.**
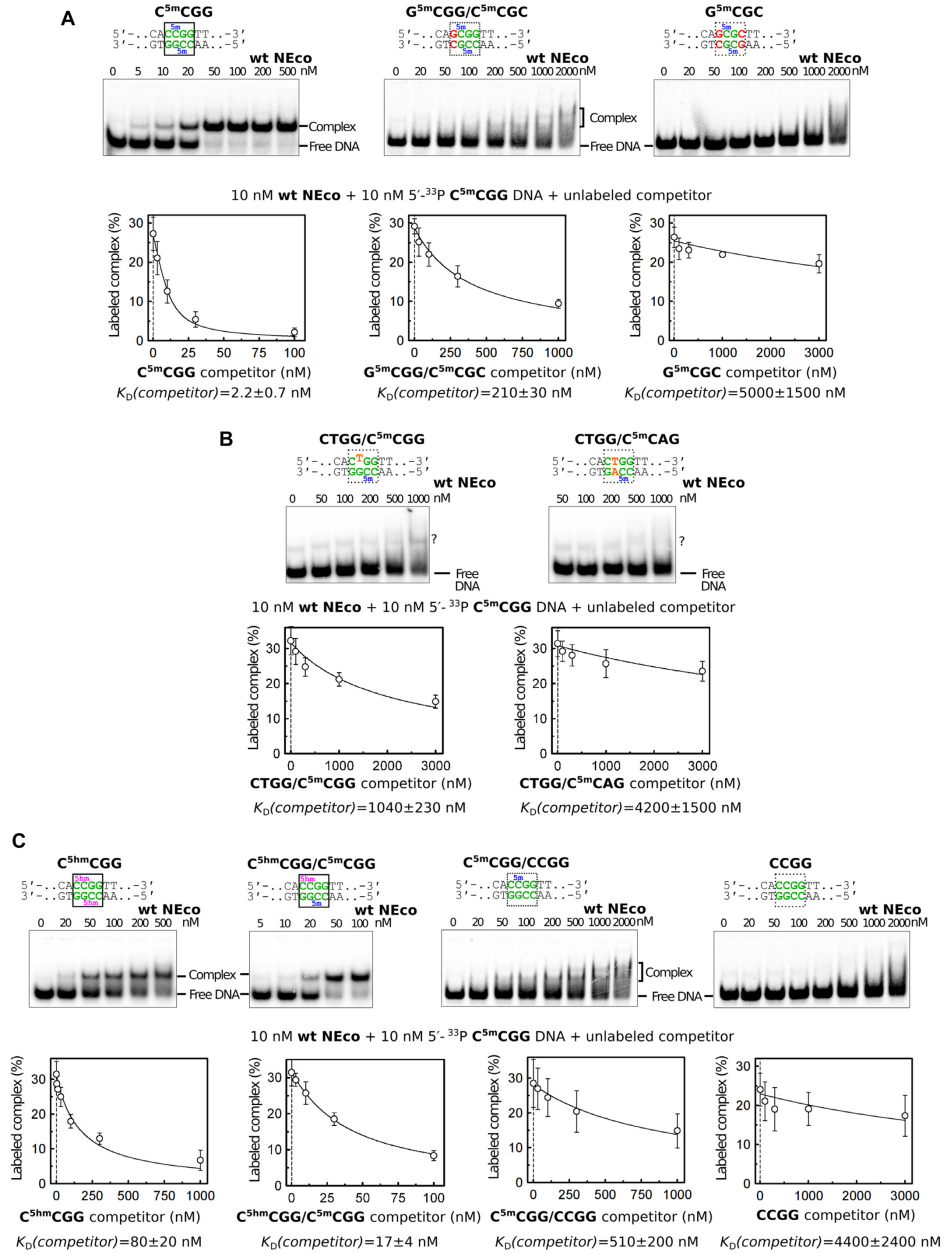
Biochemical analysis of NEco specificity. (**A**) Specificity for the outer bases. (**B**) Specificity for the inner bases. (**C**) Effect of cytosine modifications. Top of each panel: electrophoretic mobility shift assay (EMSA) with radiolabeled 30 bp DNA oligoduplexes containing variants of the NEco target sequence. 10 nM DNA and indicated NEco concentrations were used. *K*_D_ values from ≥3 independent experiments are reported in Supplementary Table S5. Bottom of each panel: EMSA-based competition experiments. 10 nM NEco, 10 nM radiolabeled C^5m^CGG DNA, and variable amounts of unlabeled competitor DNA were used. The plots show the amount of the radiolabeled protein–C^5m^CGG DNA complex as a function of unlabeled competitor concentration. *K*_D_(competitor) values were determined by non-linear fit of the competition model (solid lines) and are presented as the optimal fit value ± SE (*n* ≥ 3). Representative EMSA competition gel images are shown in Supplementary Figure S4.

**Figure 6.**
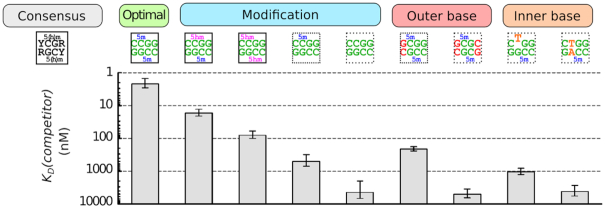
NEco specificity. *K*_D_(competitor) values were determined using the EMSA-based competition assay and DNA containing variants of the C^5m^CGG NEco target sequence (see also Figure 5 and Supplementary Figure S4). Tightest binding (lowest *K*_D_) is observed with the fully methylated C^5m^CGG DNA. Lack of cytosine modifications and replacements of the outer or inner bases impair NEco binding to various extents.

In the EMSA experiment, stable complexes were only observed with the C^5m^CGG sequence containing oligoduplexes. Affinities of the duplexes in the different groups were also determined in a competition assay by quantifying the amount of DNA required to displace the C^5m^CGG duplex. The competition assay results indicated an ∼100-fold preference of NEco for C^5m^CGG over G^5m^CGG/C^5m^CGC duplexes, and at least a 25-fold preference for G^5m^CGG/C^5m^CGC over G^5m^CGC duplexes. The result confirms that NEco recognizes the Y^5m^CGR target site, and discriminates against a purine:pyrimdine base pair at the first position and a pyrimidine:purine base pair at the fourth position of the recognition site (Figures 5A and 6).

Among sequences compatible with the Y^5m^CGR consensus the optimal sequence context for NEco according to the competition assay was T^5m^CGA, closely followed by C^5m^CGA/T^5m^CGG. The C^5m^CGG sequence used before for *in vivo* and *in vitro* characterization of EcoKMcrA and NEco (4) exhibited ∼7-fold weaker binding. The finding implies that NEco prefers a thymine:adenine over cytosine:guanine at least on one side (upstream or downstream) of the fully methylated ^5m^CpG dinucleotide (Supplementary Figure S5). We attribute this preference to extra vdW contacts made by the thymine methyl groups as discussed above (Supplementary Figure S3).

5-Methylcytosine and thymine are interchangeable for the binding of some methylated DNA binding proteins, e.g. bZIP protein AP-1 (21). To test if this could be the case for EcoKMcrA, we have characterized NEco binding to two additional DNA variants, with a single ^5m^C replaced with a thymine (resulting in a T–G mismatch, CTGG/C^5m^CGG), and with one ^5m^C:G base pair replaced by a T:A base pair (no mismatches, CTGG/C^5m^CAG). Using EMSA-based competition assay we found that both replacements dramatically decreased the affinity of NEco to DNA (∼500-fold for the T:G mismatch substrate and >2000-fold for the T:A substrate, Figures 5B and 6). This confirms the relevance of the base-specific NEco contacts to the inner ^5m^C:G base pairs observed in the structures (Figure 3 and Supplementary Figure S3).

### Biochemical characterization of the NEco methylation specificity

Earlier EMSA experiments have shown that NEco prefers fully over hemi-methylated DNA, and hemi- over non-methylated DNA (4). This implies that both methyl groups contribute to the affinity of the domain to DNA. Here, we used the competition assay to quantify the contributions of the first and second methyl groups in the C^5m^CGG context. The data indicate an ∼230-fold preference for fully over hemi-methylated DNA and ∼10-fold preference for hemi- over non-methylated DNA. We also find that NEco has a significant (∼40-fold) preference for fully methylated over fully hydroxymethylated DNA, and intermediate affinity to hybrid methylated/hydroxymethylated DNA (Figures 5C and 6).

### *In vitro* validation of the NEco DNA binding mode

**Figure 7.**
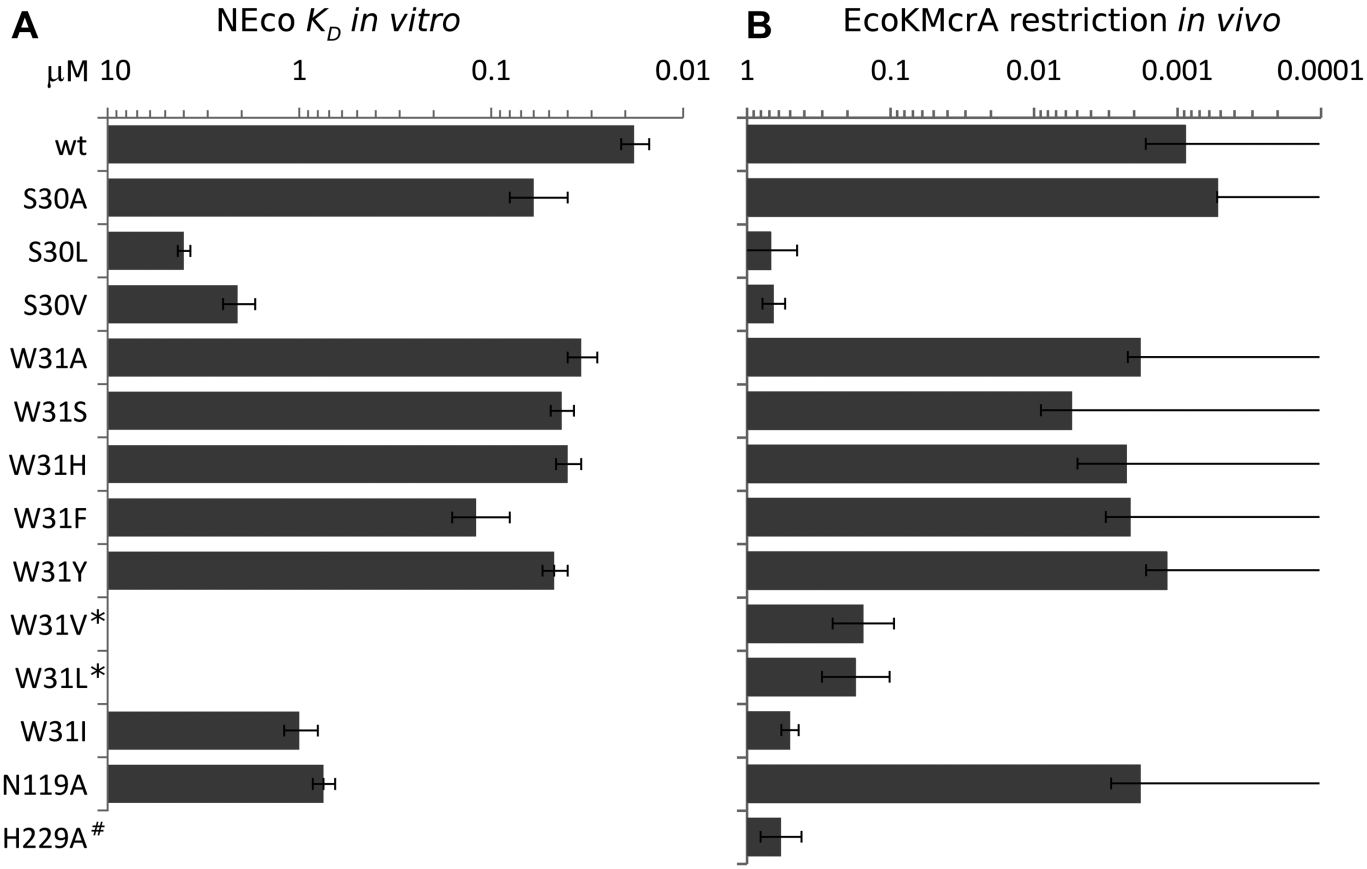
*In vitro* and *in vivo* activity of the NEco variants. (**A**) EMSA assay. The experiments were performed under standard conditions (≥3 independent experiments, 10 nM DNA and variable protein concentrations). *K*_D_ values for each experiment were calculated as described in Supplementary Methods. Average *K*_D_ values ± 1 SE are plotted. (**B**) Restriction assay. *E. coli* strains harboring wild type EcoKMcrA or its variants were transformed either by an M.HpaII ORF containing (and hence M.HpaII methylated) or control plasmid. A small colony ratio (M.HpaII/empty) indicates effective restriction. When no colonies were observed, an upper limit of one colony was used for the calculation. Error bars indicate standard deviations from three independent experiments. *No quantifiable protein-DNA complexes were formed and thus *K*_D_ could not be determined. ^#^Not tested. The catalytic H229 is located in the HNH domain and thus cannot be mutated in the isolated NEco domain.

The crystal structure suggests an involvement of S30, N119 and W31 as the key residues of the proximal and distal strand (hydroxy)methyl binding pockets. In order to test the relevance of the crystallographically observed binding mode in solution, we introduced single amino acid substitutions, designed to either expand or occlude the pockets. As we expected W31 to play a very prominent role in the binding of modified bases, a wider range of alternative residues was probed for this than for the other pocket residues. We have confirmed the structural similarity of the variants to the ‘wild type’ NEco (wt NEco) by circular dichroism and verified their properties *in vitro* by the EMSA assay (Supplementary Figures S6 and S7). The W31A, W31S, W31H, W31F, W31Y and S30A NEco variants formed complexes with DNA at similar protein concentrations as the wild type NEco domain with only marginally lower *K*_D_ values (2- to 6-fold as determined by conventional gel-shift assay). In contrast, W31I, W31L, W31V, S30L and S30V substitutions significantly decreased the binding affinity of NEco to methylated DNA (>50-fold). The N119A mutation also significantly impaired the binding (∼40-fold) (Figure 7A, Supplementary Table S5).

**Figure 8.**
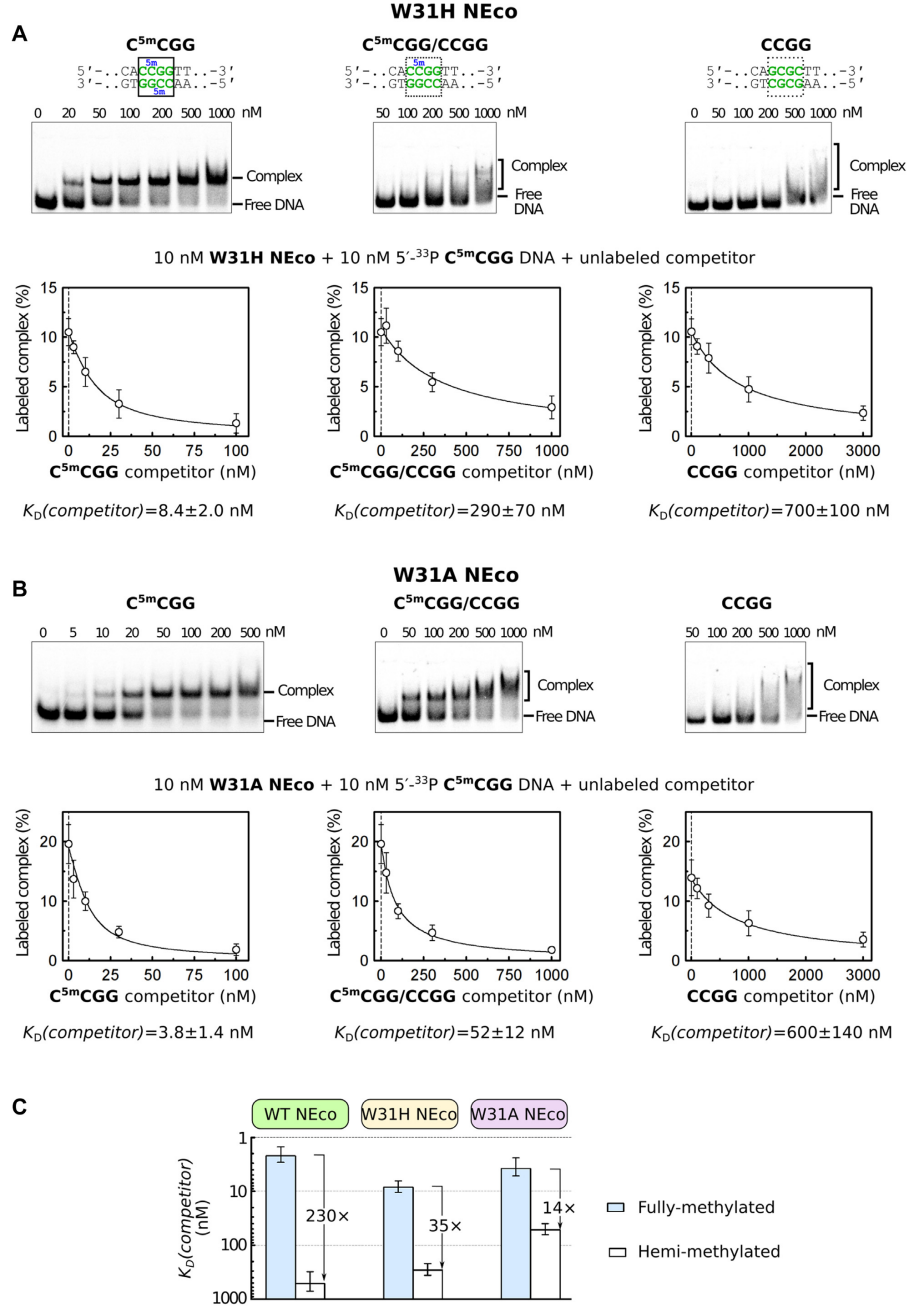
Methylation sensitivity of (**A**) W31H and (**B**) W31A NEco mutants. Top of the panels: EMSA assay with radiolabeled 30 bp DNA oligoduplexes containing fully, hemi- and non-methylated CCGG sequence. 10 nM DNA and indicated NEco variant concentrations were used. *K*_D_ values of ≥3 independent experiments are reported in Supplementary Table S5. Bottom of the panels: EMSA-based competition experiments. 10 nM NEco variant, 10 nM radiolabeled C^5m^CGG DNA, and variable amounts of unlabeled competitor DNA were used. The plots show the amount of the radiolabeled protein–C^5m^CGG DNA complex as a function of unlabeled competitor concentration. *K*_D_(competitor) values are determined by non-linear fit of the competition model (solid lines) and presented as the optimal fit value ±SE (*n* ≥ 3). Representative EMSA competition gel images are shown in Supplementary Figure S8. (**C**) Comparison of fully and hemi-methylated DNA binding by NEco and its W31H and W31H variants.

NEco variants preserved preference for fully over hemi-methylated DNA. This argues that both ^5m^C binding pockets remain at least partially functional even upon such drastic replacements as W31A. Interestingly, the ability to discriminate between fully and hemi-methylated DNA varied among mutants. For example, NEco W31H retained a strong preference for fully over hemi-methylated DNAs, as demonstrated by the competition assay. However, this preference was less pronounced than observed for the wt NEco (∼35- versus 230-fold). Qualitatively similar results were observed for other replacements of W31 with aromatic residues (W31Y, W31F). The preference for fully versus hemi-methylated DNA was further reduced in the case of NEco W31A (to ∼14-fold according to the competition assay). This variant, unlike wt NEco, forms discrete complexes with hemi-methylated DNA. Qualitatively similar results were observed for the W31S variant (Figure 8 and Supplementary Figure S7).

### Validation of the EcoKMcrA DNA binding mode by the restriction assay

Functional consequences of the pocket alterations were also tested in the context of the full-length enzyme. This was done by the restriction assay, which exhibits the modification dependence of the enzyme much more clearly than the weak *in vitro* activity (4).

We first checked for possible (unintended) acquisition of activity towards non-target DNA. For this purpose, we compared transformation efficiencies of the wt and variant EcoKMcrA expression plasmids into a dcm^−^*E. coli* strain. Transformation efficiencies varied <3-fold in side-by-side experiments (Supplementary Figure S9). Hence, we conclude that none of the variants acquired substantial activity on non-methylated DNA.

Next, we checked for possible impairment of activity towards target DNA. In the first step, BL21(DE3) cells, which lack endogenous EcoKMcrA (like other *E. coli* B strains), were transformed with pLATE31 plasmid borne expression constructs for EcoKMcrA wild type (positive control), its variants of interest, or the catalytically impaired H229A variant (negative control). The different strains were then separately made competent. In the second step, we transformed either an expression construct for M.HpaII (C^5m^CGG methylated from its production) or the parental plasmid (without C^5m^CGG methylation) into the strains, and selected for the presence of both plasmids. In order to make the assay as sensitive as possible, expression of EcoKMcrA or variants was kept minimal by glucose repression throughout the selection step. Transformation by non-methylated plasmid (without M.HpaII gene) resulted in a high colony number for all strains. A similar colony number was observed for the strain harboring inactive H229A EcoKMcrA variant, but barely any colonies were detected for the strain harboring the wild-type enzyme. As expected, the fraction of colonies obtained for methylated compared to non-methylated plasmid (or the decadic logarithm of this ratio) was anti-correlated with the methylation-specific EcoKMcrA variant activity reported before (4) (Figure 8B and Supplementary Figure S10).

The proximal methyl binding pocket is formed by S30 and N119. When N119 was replaced by alanine, the methylated plasmid restriction was not significantly impaired. In contrast, the change of S30 to valine or leucine essentially abolished EcoKMcrA restriction activity. The distal methyl binding pocket prominently features the indole ring of W31. When this residue was replaced by an aromatic residue (H, F, Y), the enzyme behaved like the wild type in our assays and no colonies were obtained. The enzyme remained functional also when W31 was replaced by alanine, and retained at least partial activity after W31S substitution. When a bulky non-aromatic residue (V, L, I) was placed instead of W31, restriction was strongly impaired, but not entirely abolished. Overall, the restriction activity correlated very well with the affinity of the NEco domain variants to DNA, with the N119A mutant as the only clear exception (Figure 8B and Supplementary Figure S10).

## DISCUSSION

### Consistency with prior biochemical and structural data

The data in this work extend prior findings on EcoKMcrA. Based primarily on EMSA experiments, the specificity was described as (Y)^5m^CGR leaving it open whether it was the result of a broader preference for ^5m^CGR averaged over two binding modes, or a genuine recognition of all four base pairs. Biochemical data in this work show that all four base pairs are specifically bound, and unambiguously confirm the Y^5m^CGR target sequence, even though structural data explain specificity only for three out of four bases in the recognition sequence.

When the structure of EcoKMcrA in the absence of DNA was solved, weak structural similarities between NEco on the one hand, and MotA, a transcription factor from T4 phage (9), and I-DmoI of the engineered I-Dre homing endonuclease fusion (8) on the other hand, were detected. Based on this similarity, surface charge and sequence conservation patterns, and results of a transposon scanning experiment, the DNA binding mode could be predicted. The crystal structure confirms the prediction of the DNA binding face, but also shows that the orientations of the DNA molecules bound to EcoKMcrA and I-DmoI and MotA differ (Supplementary Figure S11). In the light of the experimentally determined DNA binding mode of NEco, transposon insertion sites were revisited, and part of them could be given a structural interpretation (Supplementary Figure S12).

Biochemical experiments with pyrrolocytosine had indicated no change in fluorescence, which is compatible either with no base flipping, or flipping and fluorescence quenching not only in the DNA base stack, but also in the base binding pocket(s) of the protein. The lack of fluorescence changes, and the absence of obvious pockets led us to predict that EcoKMcrA does not flip DNA bases (4). The structures in this work confirm this prediction, and illustrate in detail how modified bases are recognized in the context of double-stranded DNA.

### Methyl- or hydroxymethyl binding pockets

In double-stranded DNA, the 5-methyl groups of ^5m^C bases are located on the outermost edges of the major grove. In ^5hm^C bases, there is some rotational freedom to place to the hydroxyl groups, but in the EcoKMcrA co-crystal structures, the OH groups are positioned in defined locations in the outer major groove. As the (hydroxy)methyl groups are far apart (∼8 Å away in the NEco-bound DNA), the two modifications must be recognized separately, in distinct pockets that we have termed the proximal and distal pocket, according to the base that they bind.

The EcoKMcrA proximal pocket is formed largely by the side chains of S30 and N119. The relevance of this pocket in solution is demonstrated by the detrimental effect of the S30V and S30L substitutions. These are predicted to introduce extra bulk into the pocket, and indeed, they impair DNA binding and *in vivo* activity of EcoKMcrA. In contrast, the S30A substitution, which preserves the proximal pocket, has only a mild effect on NEco and EcoKMcrA function (Figure 7 and Supplementary Figure S7).

The EcoKMcrA distal pocket has W31 at its base and T121 in the side wall. The relevance of this pocket in solution is demonstrated by the detrimental effect of substitutions that increase the steric bulk and occlude the pocket (W31V, V31L and W31I variants). As expected, NEco tolerates well exchanges of W31 to other aromatic amino acids (phenylalanine, tyrosine, and histidine). Surprisingly, however, the interaction with an aromatic side chain at the bottom of the pocket does not seem to be essential. When W31 is replaced by small residues (alanine and serine), the effect is very mild.

EcoKMcrA is generally considered as a barrier for methylated DNA. However, the enzyme is also effective against hydroxymethylated DNA (unless it is further glucosylated) (4). The structure with T^5hm^CGA DNA readily explains this observation (Figure 4). In both proximal and distal strands, the hydroxymethyl groups are accommodated in the respective pockets and form direct hydrogen bonds with the protein backbone. Despite these extra H-bonds the fully hydroxymethylated DNA is bound by NEco approx. 40-fold weaker than fully methylated DNA (2 nM versus 80 nM *K*_D_ according to competition assay, Figure 5C), suggesting that the affinity of NEco to modified cytosine variants is primarily governed by other factors, such as different dehydration free energies of ^5m^C and ^5hm^C. Nevertheless, the remaining affinity to fully hydroxymethylated DNA must be sufficient for restriction.

### Cooperative methyl group recognition

It has been known already prior to this work that EcoKMcrA had a preference for fully over hemi-methylated, and for hemi- over non-methylated DNA. Such specificity makes sense, because the DNA of both incoming phage and the *E. coli* host (e.g. a human) are expected to be methylated on both strands. The surprising finding of this study is that the second methyl group adds more to affinity than the first one. For independent methyl group binding sites, this observation is counterintuitive. If one methyl binding pocket provides a ‘better’ methyl environment than the other, the first methyl group should bind in the ‘better’, and the second one in the ‘worse’ site, especially if the modified sequence is palindromic. The high gain in affinity with addition of the second methyl group may be attributable to cooperativity. Interestingly, the cooperativity is preserved in the W31H variant, but lost in the W31A variant (Figure 8), suggesting that an aromatic residue at the position 31, optimally a tryptophan, is required for cooperative binding of two methylated cytosines. Unfortunately, the crystal structures do not provide an obvious explanation for the cooperativity, since there is little change between the DNA-free and -bound NEco domains (Supplementary Figure S2 and Supplementary Table S3).

### Comparison with other domains binding modified cytosine bases in DNA

Modified cytosine binding domains typically function as monomers and bind DNA asymmetrically, even when the target sequence is palindromic. Broadly, they can be grouped into domains that flip a modified base and domains that recognize cytosine modifications in the context of double-stranded DNA (Supplementary Figure S13). Within each group, the precise modification requirements can vary, and phylogenetically related proteins can bind ^5m^C, ^5hm^C or even ^g5hm^C.

The first group of domains is represented in prokaryotes and eukaryotes. It is dominated by SRA domains, now recognized as a specific subfamily of PUA domains (22). The group also contains domains of PUA unrelated fold, for example the N-terminal domain of EcoKMcrBC (23). The domains in this group contain only a single pocket for a modified base. Bases in regular double-stranded DNA cannot reach it, and a modified base has to be extruded from the double helix to be scrutinized in the pocket (10–12,24). If modified cytosine bases are present in the ‘other’ strand, they tend to go undetected, or are detrimental for binding (24,25). For some SRA domains, it has been explicitly shown that binding to hemi-methylated DNA is the main physiological function (26). Although many isolated domains in this group bind hemi-modified DNA as well or better than fully modified DNA, proteins containing such domains frequently dimerize via fusion to other dimeric domains. The complete proteins, typically from prokaryotes, then have a preference for DNA with more than one modification, as shown for restriction endonucleases of the TagI (27) and PvuRts1I (28–30) groups. Note that ScoMcrA contains an SRA domain for detecting modified cytosine bases, which is not homologous to NEco, despite the misleading analogy in the names of ScoMcrA and EcoKMcrA (31,32) (Supplementary Figure S14).

The second group of domains that bind modified cytosine bases is found in eukaryotes and consists of zinc fingers (e.g. Zfp57 (33), KAISO (14)), and other structurally similar domains (MBD1 (13), MBD3 (PDB code 6CC8) or MeCP2 (34)). These domains recognize methyl- or hydroxymethylcytosine in the biologically predominant CpG context. Biochemical data indicate that these domains bind better to fully than to hemi-modified DNA (35). Crystallographic data confirm that the domains in this group tend to make contacts with the methyl or hydroxymethyl groups in both DNA strands. Moreover, they show that the contacts are made without flipping of DNA bases. In all cases, the cytosine modifications stack against the guanidino groups of arginine side chains, frequently salt-bridge anchored by acidic residues (Supplementary Figure S13).

The NEco domain of EcoKMcrA detects cytosine modifications more like proteins in the second than in the first group. In particular, the enzyme does not flip modified DNA bases. Moreover, it has a clear preference for fully over hemi-modified DNA. However, similarity ends there. NEco and domains of the second group are neither sequentially nor structurally similar. The latter accommodate methyl or hydroxymethyl groups in pockets that have side walls contributed by arginine side chains. In contrast, NEco accommodates methyl or hydroxymethyl groups in uncharged pockets. Amino acid substitutions that occlude these pockets severely impair binding of NEco to fully modified DNA. Other mutations are surprisingly well tolerated, even when they drastically alter the pocket properties. The biophysical principles that underlie this unexpected robustness of modification detection remain to be elucidated.

## DATA AVAILABILITY

The raw diffraction data were deposited at the REPOD database (http://dx.doi.org/10.18150/repod.1407597). The final model coordinates and the corresponding structure factors were deposited in Protein Data Bank under accession codes: 6R64 (C5mCGG), 6T21 (T5mCGA) or 6T22 (T5hmCGA).

## Supplementary Material

gkz1017_Supplemental_File
