# Supplementary material for: Crystal structure of the EcoKMcrA N-terminal domain (NEco): recognition of modified cytosine bases without flipping
Source: Nucleic Acids Res. 2019 Nov 14;47(22):11943–55. doi: 10.1093/nar/gkz1017 (PMC7145662; doi:10.1093/nar/gkz1017)
Supplement: gkz1017_Supplemental_File [file gkz1017_supplemental_file.pdf]

Supplementary material for:

Crystal structure of the EcoKMcrA N-terminal domain (NEco): recognition of modified cytosine bases without flipping

Anton Slyvka[1], Evelina Zagorskaitė[2], Honorata Czapinska[1], Giedrius Sasnauskas[2,#], Matthias Bochtler[1,3,#]

[1]*International Institute of Molecular and Cell Biology, Trojdena 4, 02-109 Warsaw, Poland*

[2]*Institute of Biotechnology, Vilnius University, Saulėtekio av. 7, 10257 Vilnius, Lithuania*

[3]*Institute of Biochemistry and Biophysics PAS, Pawinskiego 5a, 02-106 Warsaw, Poland*

Supplementary Methods

Calculation of the K_D values

If s_0 and s denote total and free DNA concentrations, and p_0 and p denote total and free protein concentrations, then the concentration of the complex is $s_0 – s = p_0 – p$. The binding equilibrium can then be written as $K_D = s \times [p_0 – (s_0 – s)]/(s_0 – s)$. This quadratic equation in s can be rearranged to:

$$s=\{s_0 – p_0 – K_D + [(s_0 + p_0 + K_D)^2 – 4 \times s_0 \times p_0]^{0.5}\}/2$$

Dissociation constants K_D were determined by fitting the dependence of the free DNA concentration s on the total protein concentration p_0. Values reported in Table 1 are average K_D values determined from 3 or more independent experiments ±1 SE.

EMSA competition experiments

The EMSA samples contained NEco protein (final concentration 10 nM), radiolabeled $C^{5m}CGG$ or $T^{5m}CGA$ DNA (10 nM), and variable amounts (final concentrations 3-3000 nM) of unlabeled competitor DNA carrying various recognition site variants. Electrophoresis was run as described previously (1). The amounts of the radiolabeled protein–DNA complex (i.e., the complex with either $C^{5m}CGG$ or $T^{5m}CGA$ DNA) at different competitor concentrations were determined by densitometric analysis of EMSA gel images using OptiQuant software (Packard Instrument). Competition data were analyzed as described earlier (2), providing dissociation constant for the protein—radiolabeled DNA complex K_D*(labeled)*, and for the protein—unlabeled competitor DNA interaction K_D*(competitor)*. All competition experiments were repeated at least 3 times. The reported K_D*(competitor)* values are the optimal fit values to the combined data ±1 SE as reported by the nonlinear fitting software.

In competition experiments performed with wt NEco and labeled $C^{5m}CGG$ DNA, the determined K_D*(labeled)* was approximately equal to 20 nM; this reflects the fact that under our experimental conditions (10 nM radiolabeled DNA and 10 nM wt NEco) in the absence of the competitor, approximately 1/4 DNA and 1/4 protein formed the complex detectable in EMSA gels. In theory, if the unlabeled $C^{5m}CGG$ DNA is used as a competitor, one would expect approximately equal values of K_D*(labeled)* and K_D*(competitor)*, as in this case both constants reflect the affinity of wt NEco to the same DNA. In practice, however, the K_D*(competitor)* reported by the fitting procedure was approximately equal to 2 nM (Figure 4C and 5C), i.e. approx. 10-fold lower than the K_D*(labeled)*. Similar discrepancies were observed with other protein and DNA variants. For example, the K_D*(labeled)* values reported in the case of wt NEco and labeled $T^{5m}CGA$ DNA (Figure S3C) by the non-linear fitting procedure were 30-35 nM (correspond to approx. 20% of

complex observed with 10 nM wt NEco and 10 nM labeled $T^{5m}CGA$ DNA in the absence of competitor), but $K_D(competitor)$ for $T^{5m}CGA$ DNA is 5 nM (Figure S3C); in the case of W31H NEco mutant and $C^{5m}CGG$ DNA, $K_D(labeled)$ was approx. 75 nM (corresponds to approx. 10% of complex observed with 10 nM W31H NEco and 10 nM labeled $C^{5m}CGG$ DNA), but the $K_D(competitor)$ for $C^{5m}CGG$ DNA is 9 nM (Figure 6AB); in the case of W31A NEco and $C^{5m}CGG$ DNA, $K_D(labeled)$ was approx. 30-50 nM (corresponds to approx. 15-20% of complex observed with 10 nM W31A NEco and 10 nM labeled $C^{5m}CGG$ DNA), but the $K_D(competitor)$ for $C^{5m}CGG$ DNA is 4 nM (Figure 6CD). We presume that these discrepancies primarily arise due to partial dissociation of the protein-DNA complexes during the electrophoretic runs, resulting in a reduced amount of the cognate complex in the absence of the competitor, and, consequently, a higher $K_D(labeled)$ value; the second contributing factor may be inaccuracies in the active protein and DNA concentrations. Nevertheless, the EMSA-based competition experiments allowed us to perform quantitative analysis of the relative NEco binding affinities to the studied DNA variants, including the ones that do not form quantifiable complexes with NEco.

Supplementary Figures

Fig. S1

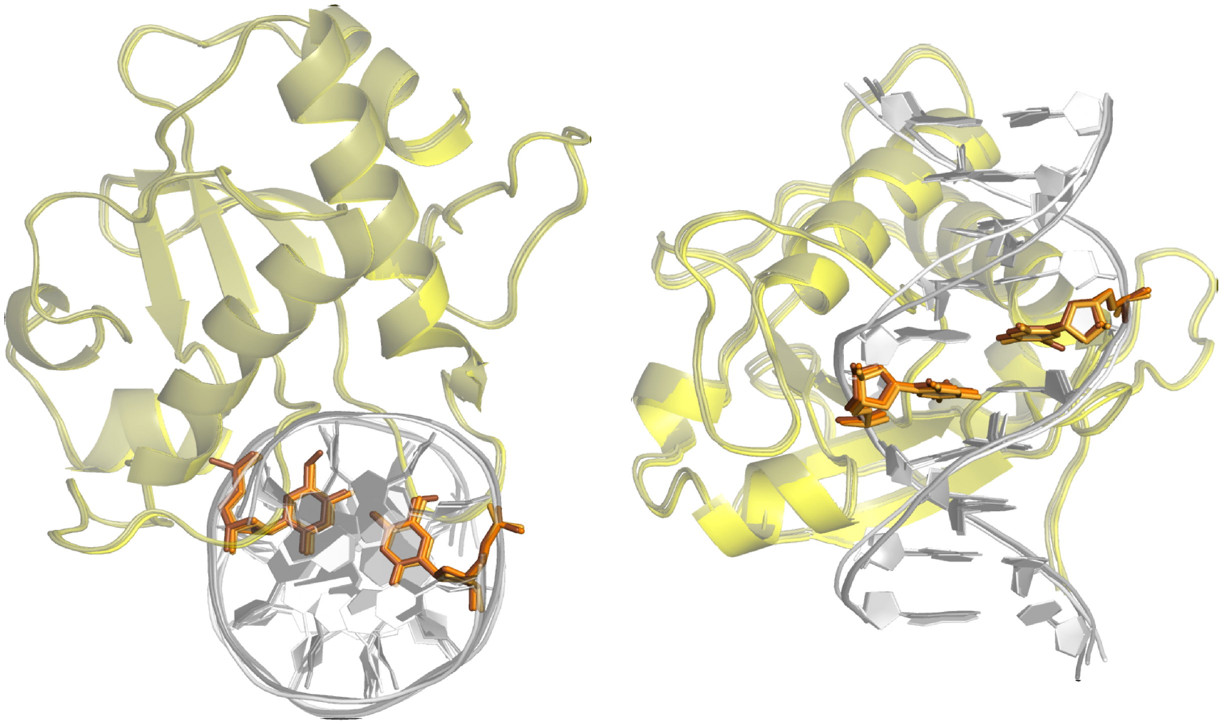

Fig. S1: EcoKMcrA N-terminal domain (NEco) DNA complexes. The crystal structures of the complexes of NEco with 10-mer DNA duplexes containing $C^{5m}CGA$, $T^{5m}CGA$ and $T^{5hm}CGA$ target sequences were overlaid to indicate the absence of major differences in the protein and DNA conformations. In each case only one of two molecules in the asymmetric unit was used for the comparison.

Fig. S2

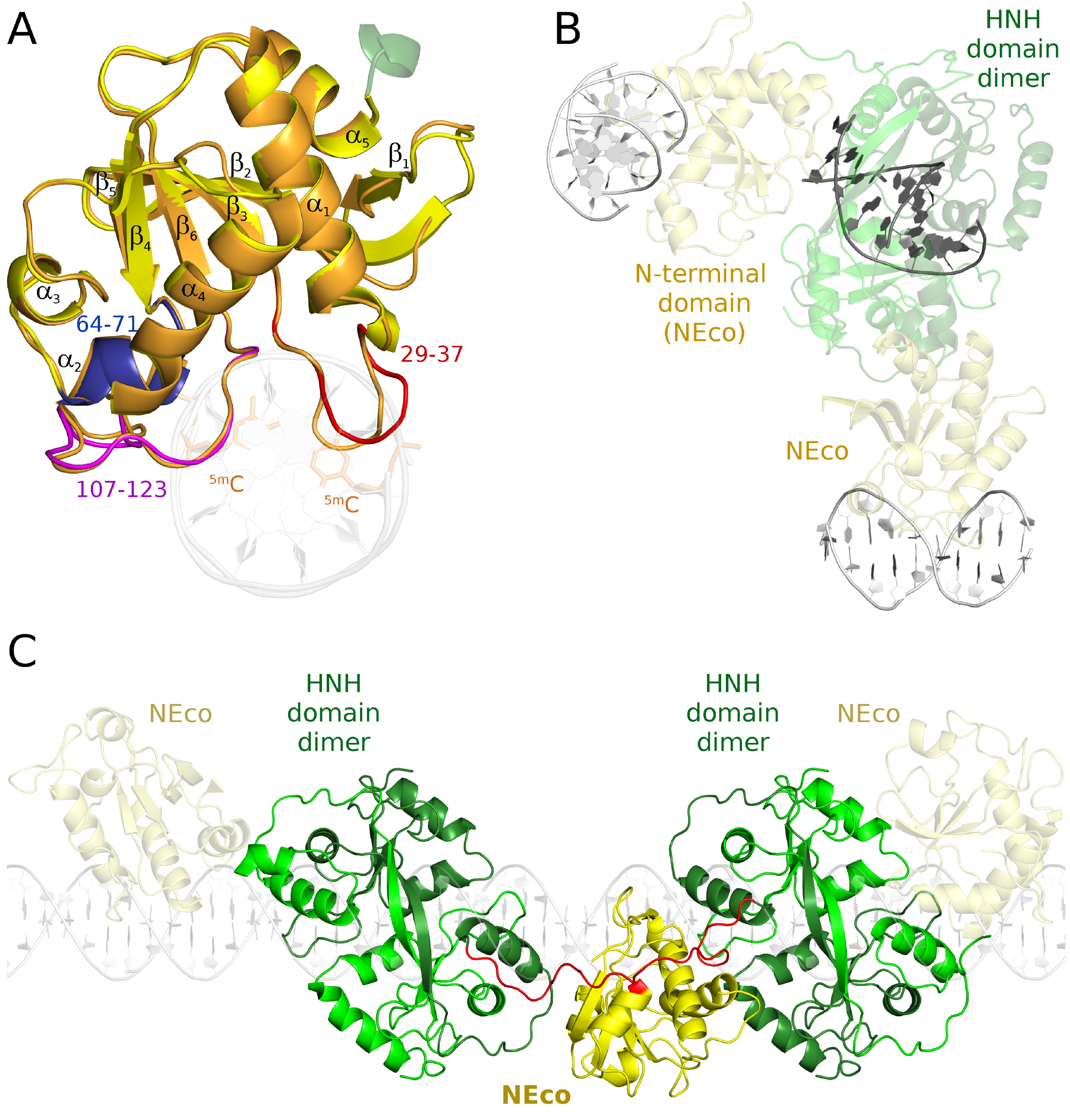

Fig. S2: EcoKMcrA-DNA interaction. (A) Overlay of the EcoKMcrA N-terminal domain (NEco) structures obtained in the presence and absence of DNA. **(B)** Model of the full-length EcoKMcrA-DNA complex preserving two crystallographically observed relative domain orientations (1). The DNA bound to the HNH domain was modeled based on the Hpy99I-DNA structure (3). The NEco domain bound DNA was based on this work. **(C)** Model of the full-length EcoKMcrA DNA complex assuming straight DNA and flexible interdomain linker (shown in red). The HNH domains were modeled 10 nt upstream and downstream of the proximal strand ^{5m}C base (counted to the central bp of the pseudopalindromic HNH domain target). The second "dangling" NEco domain is shown in faint yellow.

Fig. S3

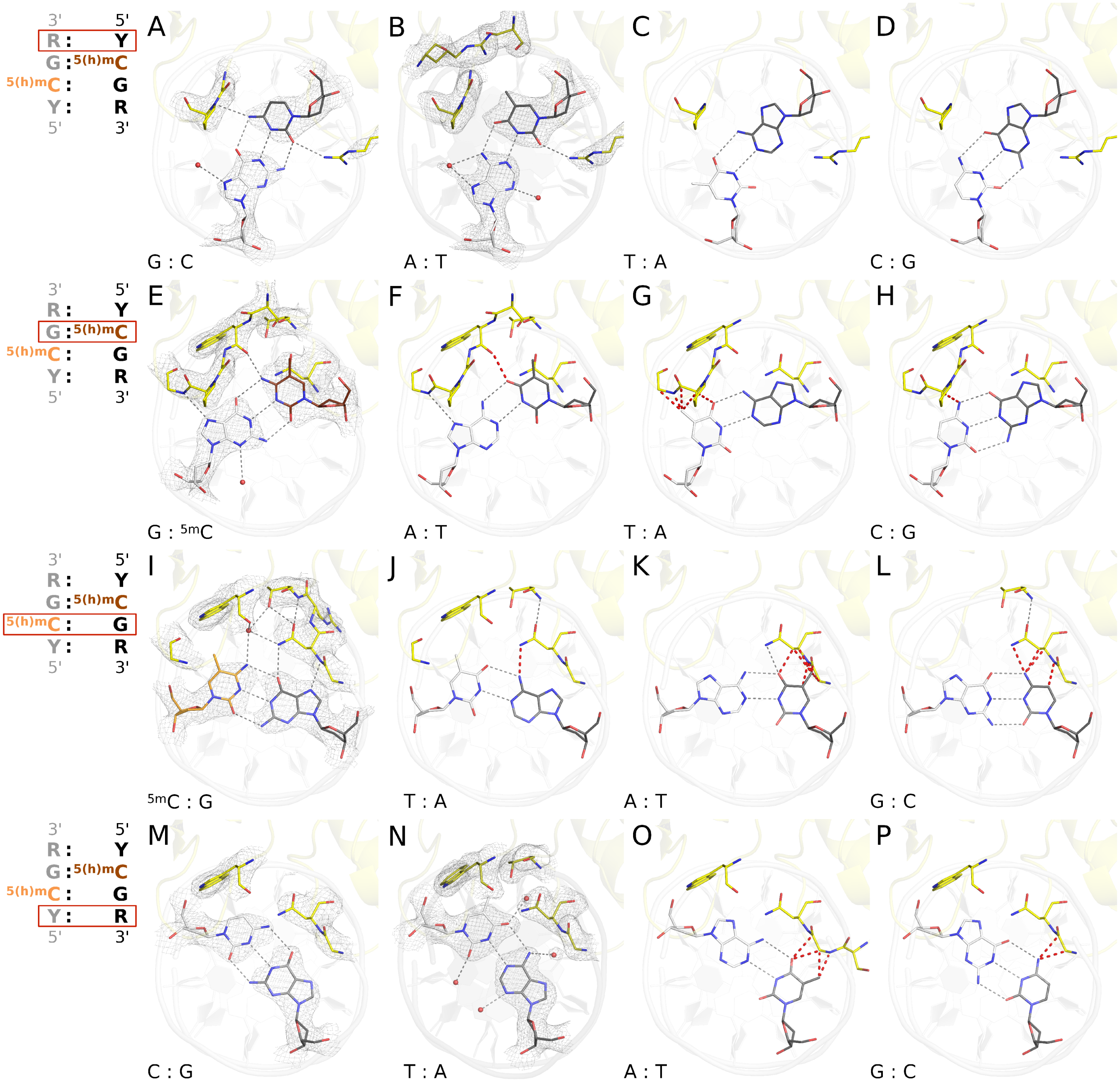

Fig. S3: Discrimination of alternative base pairs by NEco. The panels with electron density maps show the crystal structures with the target sequence following the consensus. The other panels show clashes of alternative base pairs, *in silico* modeled based on the structure of the NEco-C^{5m}CGG complex. 3.0 Å cutoff was used for clash estimation. The composite omit maps in the first column were calculated for the NEco-C^{5m}CGG structure and contoured at 1.5 rmsd, the ones in the second column for NEco-T^{5m}CGA with 1.0 rmsd contour.

Fig. S4

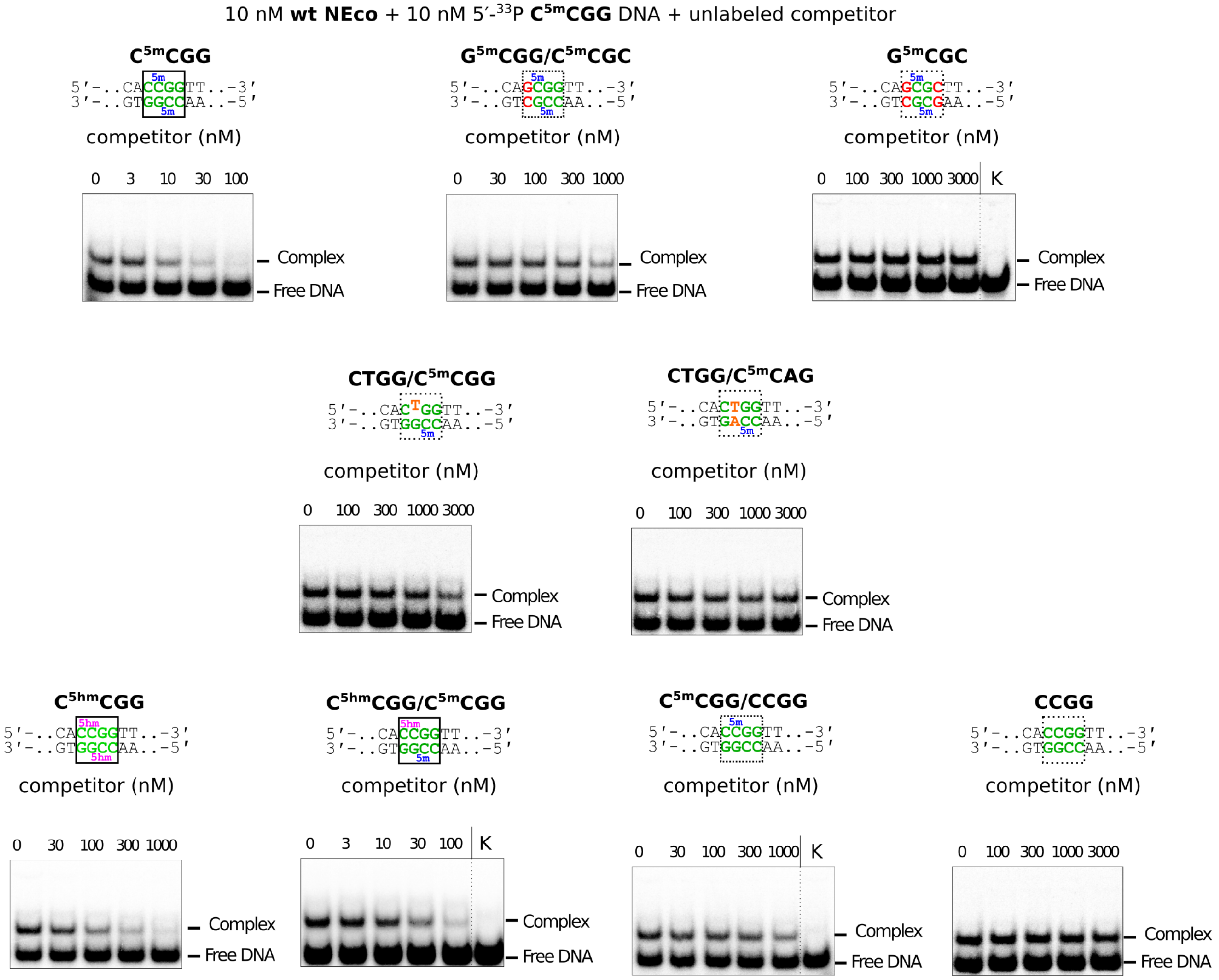

Fig. S4: Competition experiments with wt NEco. EMSA competition experiments were performed with wt NEco and various DNA variants shown above the gels. The reactions contained 10 nM radiolabeled C^{5m}CGG DNA, 10 nM wt NEco, and variable amounts of unlabeled competitor DNA (concentrations shown above gel lanes). Representative gels of at least 3 independent experiments are shown.

Fig. S5

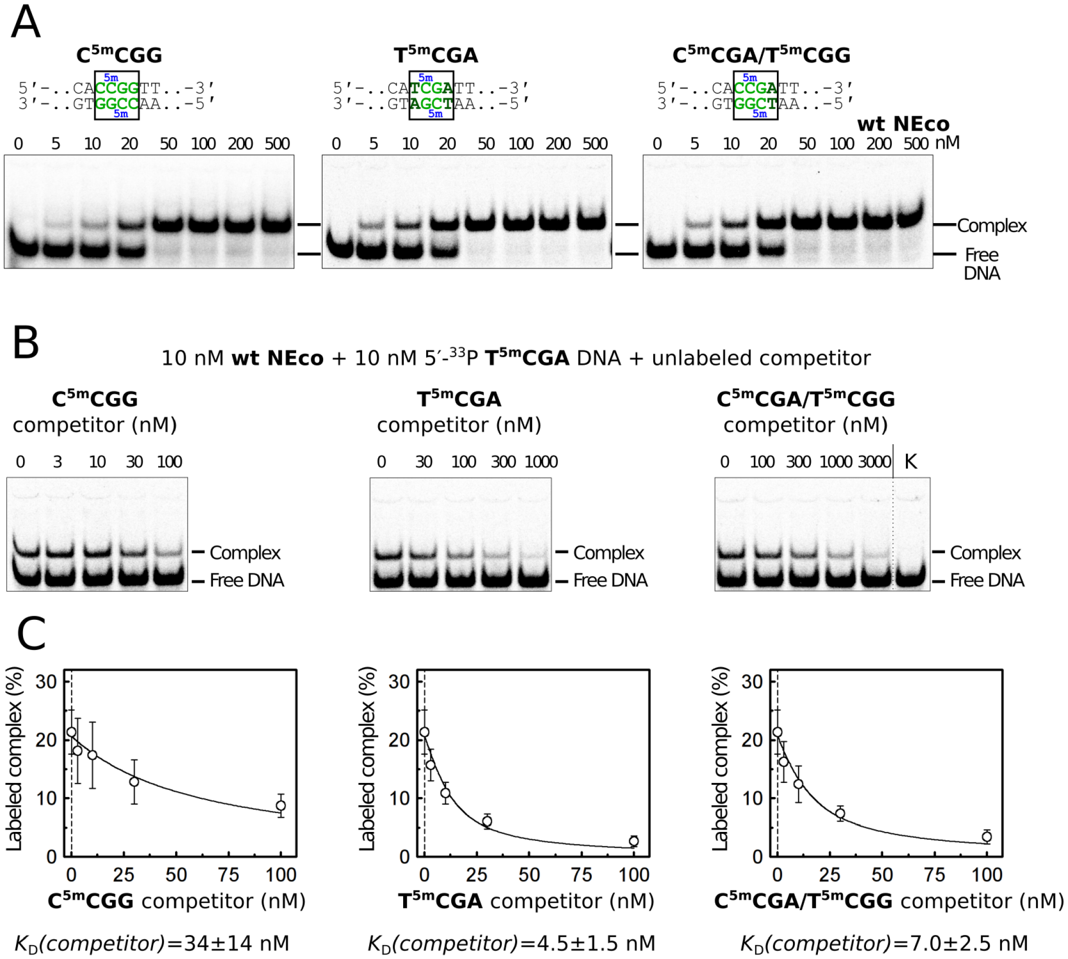

Fig. S5: NEco binding to different 5′-Y(^{5m}C)GR-3′ sequence variants. (A) Standard electrophoretic mobility shift assay (EMSA). Radiolabeled 30 bp DNA oligoduplexes contained fully-methylated CpG dinucleotide in 3 different sequence contexts: symmetric C^{5m}CGG, symmetric T^{5m}CGA and asymmetric C^{5m}CGA/T^{5m}CGG. The DNA concentration was 10 nM, concentrations of wt NEco are indicated above gel lanes. The gel with C^{5m}CGG DNA is also shown in Figure 5A. (B) EMSA-based competition experiment performed with wt NEco and C^{5m}CGG, T^{5m}CGA and C^{5m}CGA/T^{5m}CGG DNAs. The reactions contained 10 nM of radiolabeled T^{5m}CGA DNA, 10 nM of wt NEco, and variable amounts of unlabeled competitor DNA (concentrations shown above gel lanes). (C) Quantification of EMSA competition experiments. The plots show the amount of the radiolabeled protein-T^{5m}CGA DNA complex as a function of the unlabeled competitor concentration. The nonlinear fit of the competition model (solid lines) led to K_D*(competitor)* values indicated below the gels (presented as the optimal fit value ±SE, n ≥ 3).

Fig. S6

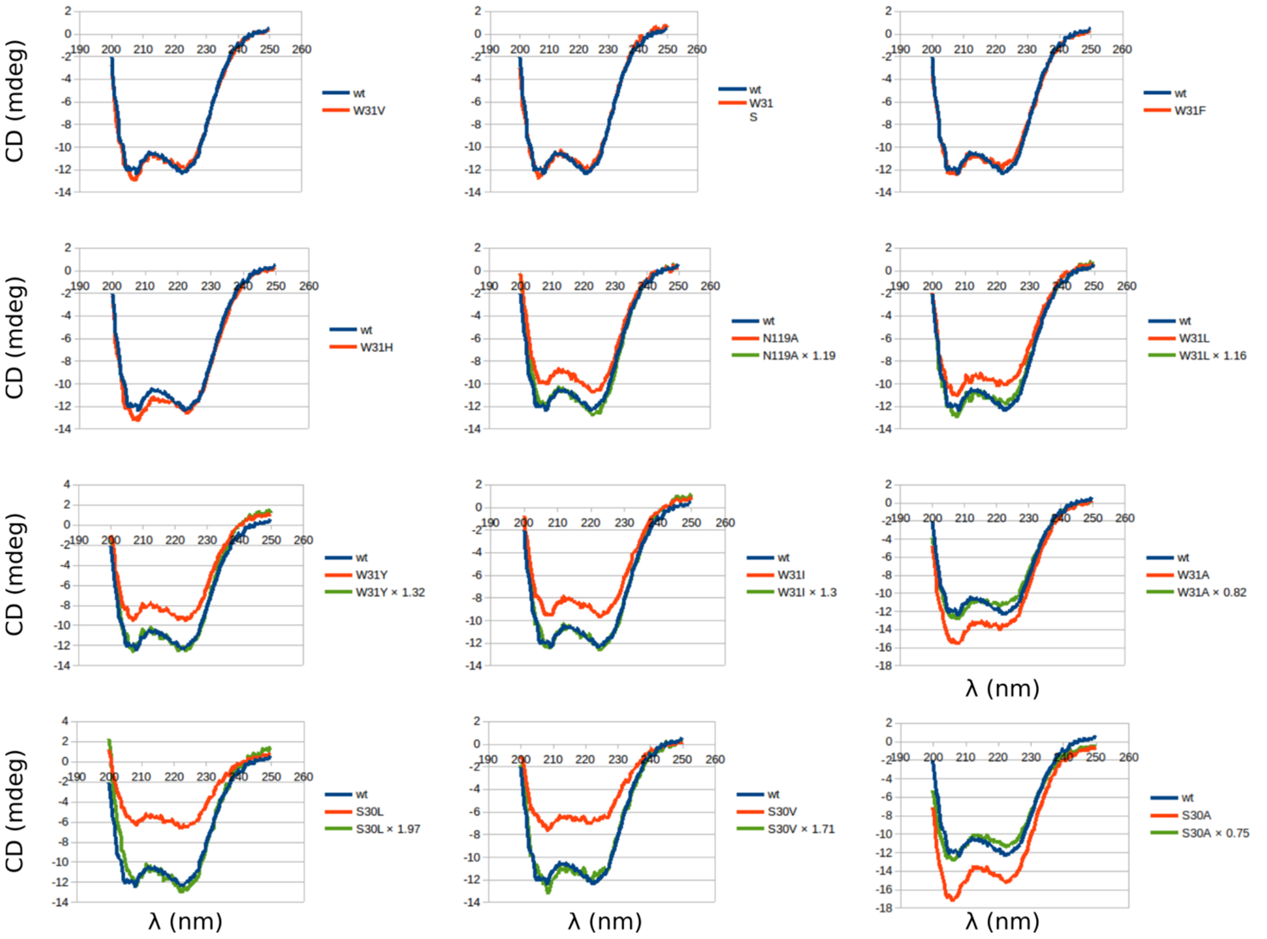

Fig. S6: Far-UV CD spectra of wt NEco and NEco variants. Circular dichroism (CD) measurements were carried out on a Jasco J-815 CD spectrometer (Jasco Corporation, Tokyo, Japan). All samples contained 10 µM protein (0.13 mg/mL) in 10 mM Tris-HCl (pH 7.5) and 25 mM NaCl. The measurements were made at 25 °C using a 1.0 mm pathlength cell. The spectra were corrected for the buffer contribution. Each graph shows the spectra of wt NEco (dark blue line), the respective mutant (red line), and, in cases where differences between the wt and mutant NEco signals are significant, the mutant spectra with the signal multiplied by a correction factor (green lines, correction factors indicated in the graph legends). Correction factor in each case was selected such that it minimized the sum of squared differences between the signals of wt and mutant spectra.

Fig. S7

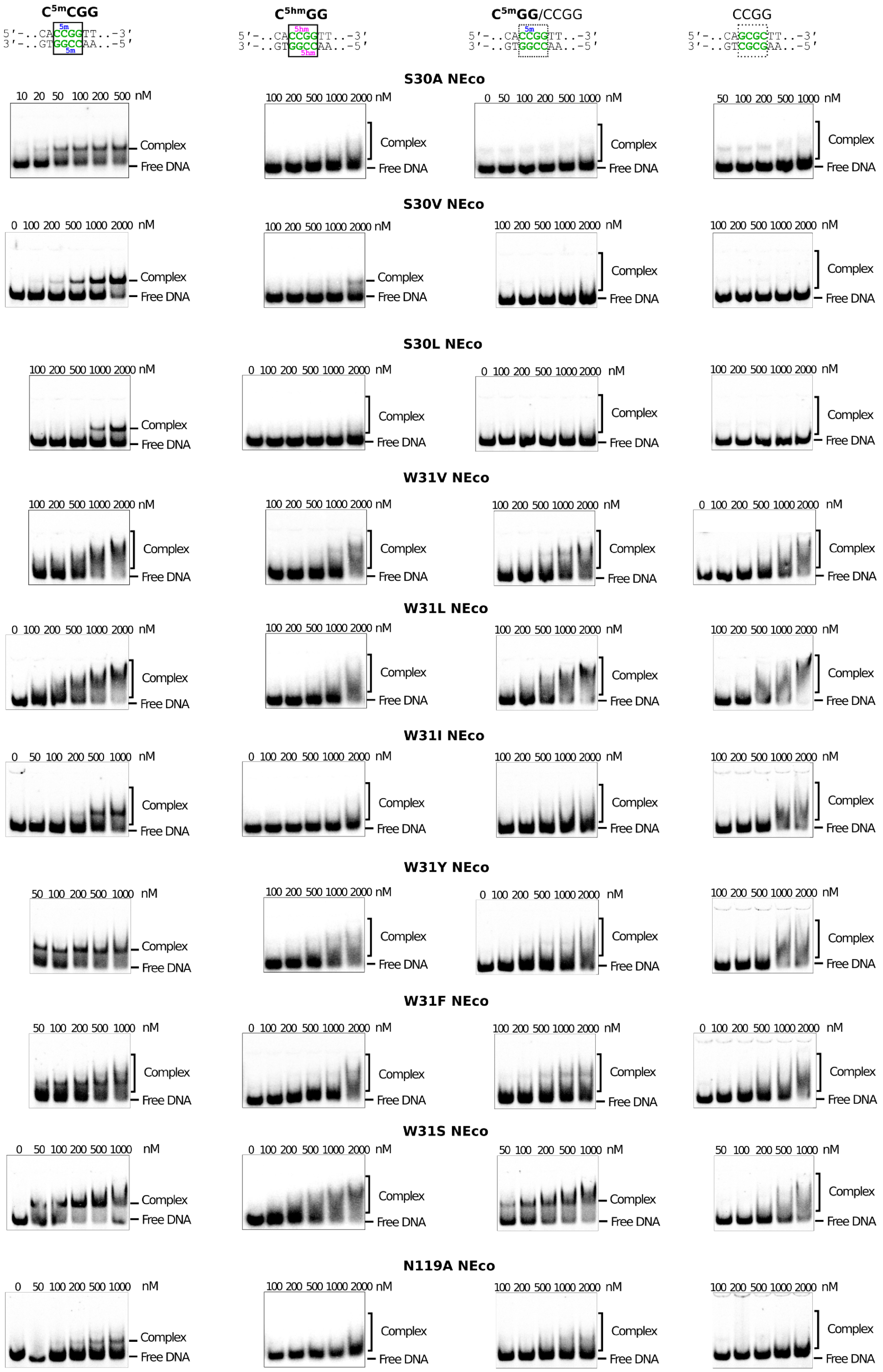

Fig. S7: The effect of pocket mutations on NEco binding. EMSA was performed with fully-methylated (C^{5m}CGG, 1st column), fully-hydroxymethylated (C^{5hm}CGG, 2nd column), hemi-methylated (C^{5m}CGG/CCGG, 3rd column), or non-methylated (CCGG, 4th column) DNA oligoduplexes and NEco mutants. The DNA concentration was 10 nM, concentrations of NEco domains are indicated above gel lanes. Calculated K_D values are reported in Table S5.

Fig. S8

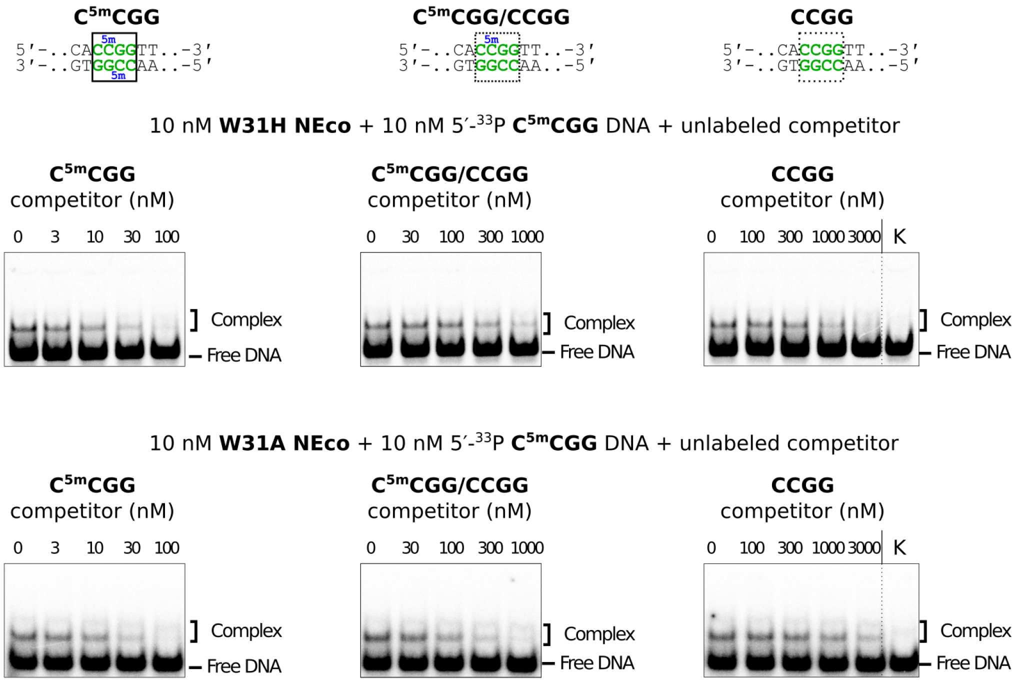

Fig. S8: EMSA-based competition experiments with NEco mutants W31H and W31A. EMSA competition experiments were performed with W31H and W31A NEco mutants and fully-methylated (C^{5m}CGG), hemi-methylated (C^{5m}CGG/CCGG), or non-methylated (CCGG) DNA. The reactions contained 10 nM radiolabeled C^{5m}CGG DNA, 10 nM NEco, and variable amounts of unlabeled competitor DNA (concentrations shown above gel lanes). Representative gels of at least 3 independent experiments are shown.

Fig. S9

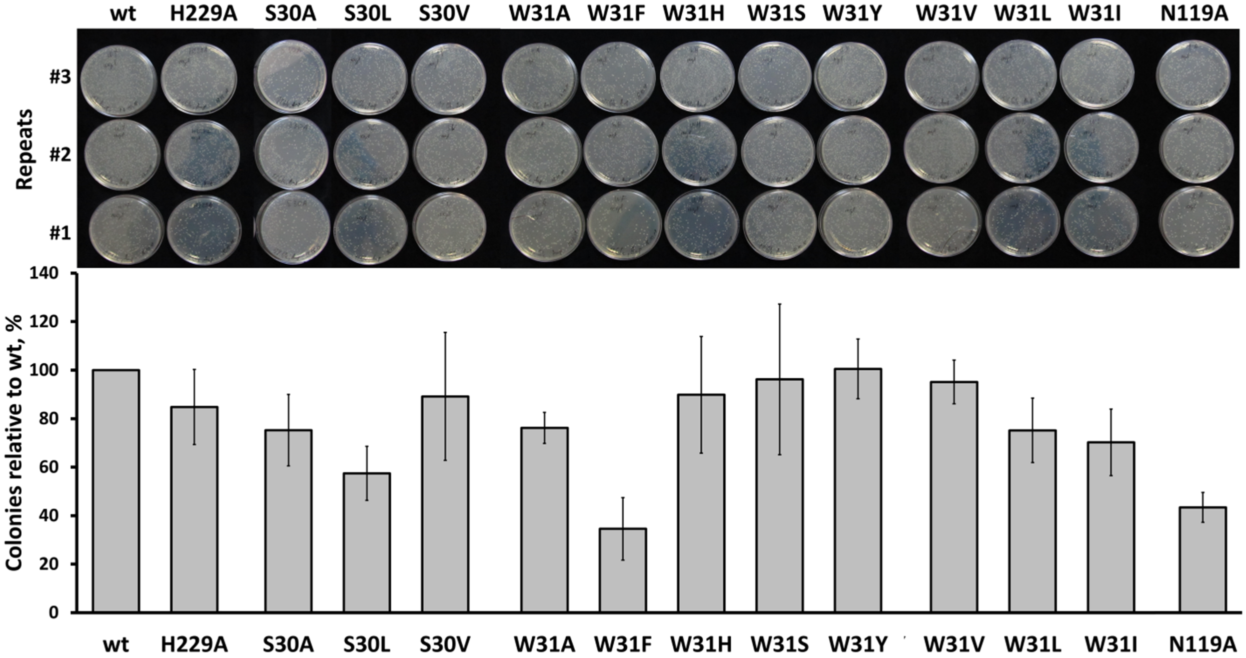

Fig. S9: Restriction activity of NEco variants in the context of the full-length EcoKMcrA. Equal amounts (18 ng) of pLATE31 plasmid carrying an open reading frame for wt EcoKMcrA, its H229A catalytic variant, or variants of interest was transformed into a BL21(DE) (mcrA$^-$) strain followed by selection for the maintenance of the plasmid. The comparable transformation rates indicate that none of the variants has acquired significant non-specific nuclease activity.

Fig. S10

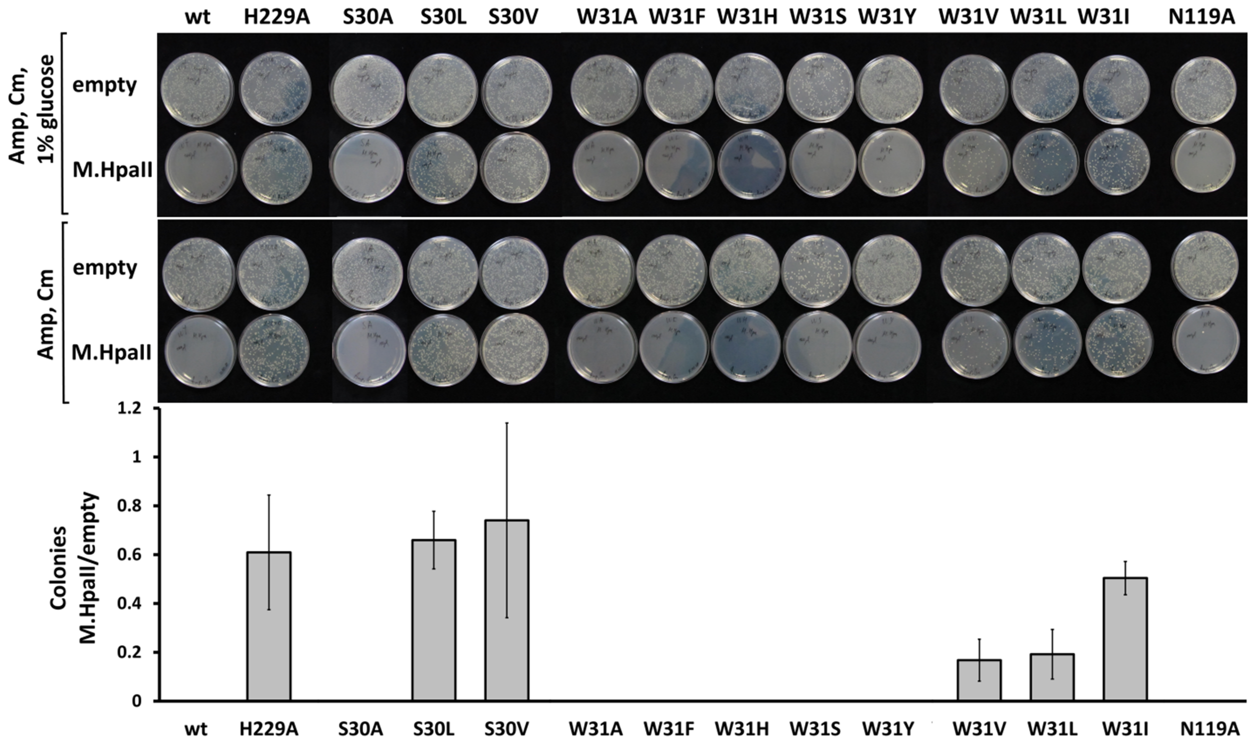

Fig. S10: Restriction activity of NEco variants in the context of the full length EcoKMcrA. BL21(DE3) ($McrA^-$) *E. coli* cells were transformed with pLATE31 plasmid carrying an open reading frame for wt EcoKMcrA, its catalytically inactive H229A variant, or variants of interest. The transformed strains were separately made competent. Competent cells were then tested for their ability to restrict pACYC184 plasmid carrying the gene for M.HpaII, which methylates DNA so that it is restricted by wt EcoKMcrA. In order to correct for variations in the efficiency of rendering the cells competent, the same strains were also transformed by empty pACYC184 (‘empty’). The ratio of the pACYC184-M.HpaII and pACYC184 colony counts was then taken as a measure of impairment of EcoKMcrA restriction activity. The data presented in this figure were also used for the logarithmic plot shown in Fig. 7.

Fig. S11

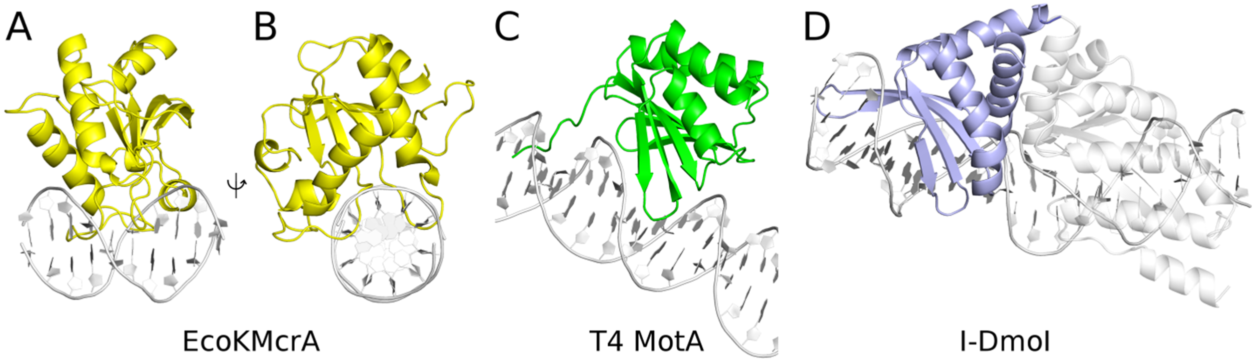

Fig. S11: Comparison of the DNA binding modes of (A) NEco, (B) T4 MotA transcription factor (4) and (C) I-DmoI homing endonuclease (5). The proteins share distant structural similarity, but bind DNA very differently. EcoKMcrA is oriented in analogous way as the two other proteins in the right panel. In the left panel the structure was 90° rotated to match the DNA orientations.

Fig. S12

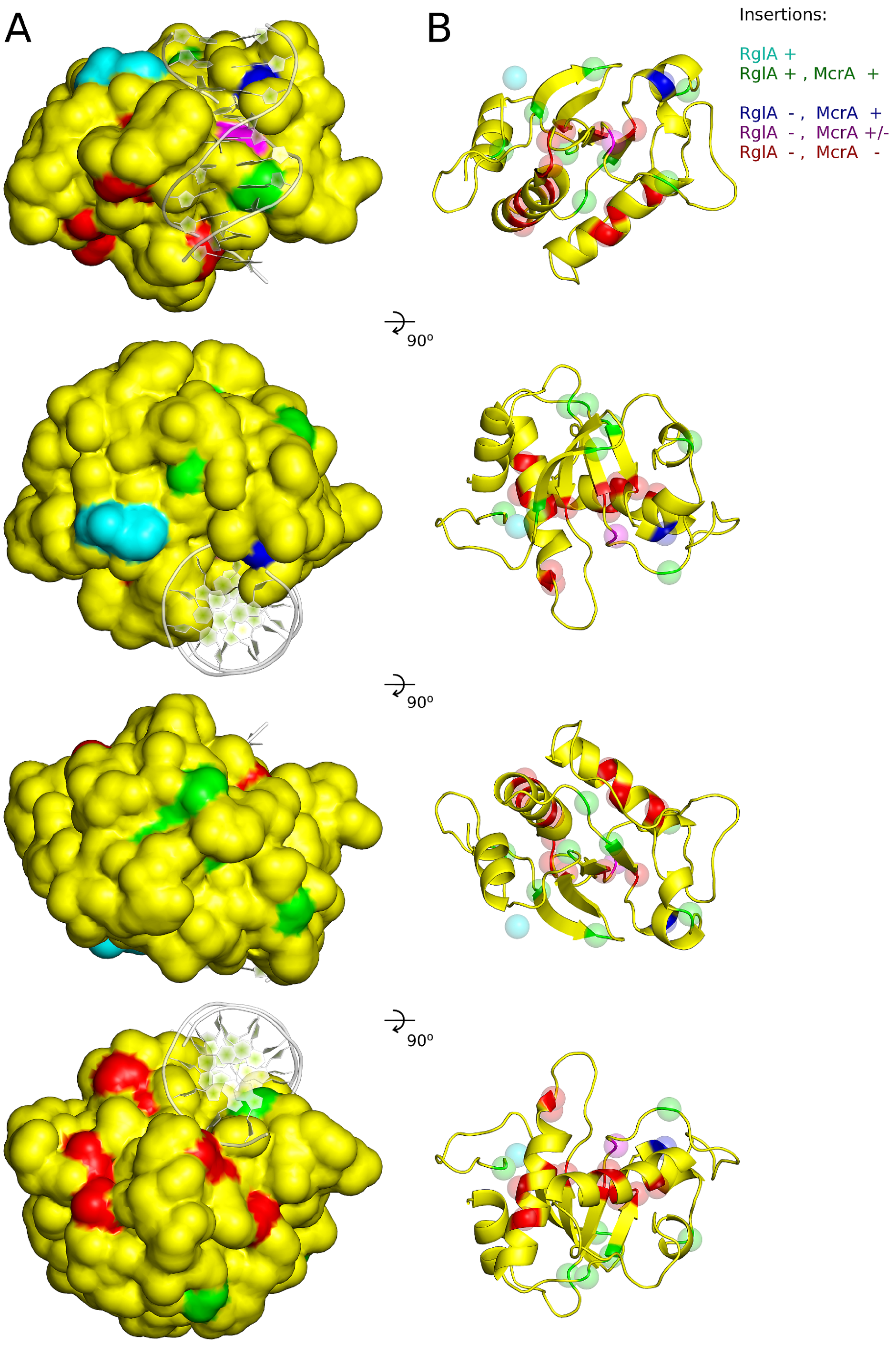

Fig. S12: Location of the insertions affecting the activity of EcoKMcrA in a transposon scanning experiment (6). The positions indicated in green and cyan have no impact on the restriction activity. The ones indicated in red, blue and magenta obliterate the RglA (^{5hm}C driven) restriction activity, and eliminate, moderately affect, or leave unaffected the McrA (^{5m}C driven) restriction activity (respectively). Locations of the insertions are indicated (A) on the surface of the protein or (B) as semi-transparent balls in the position of Cα atoms in the cartoon representation of the Cα trace.

Fig. S13

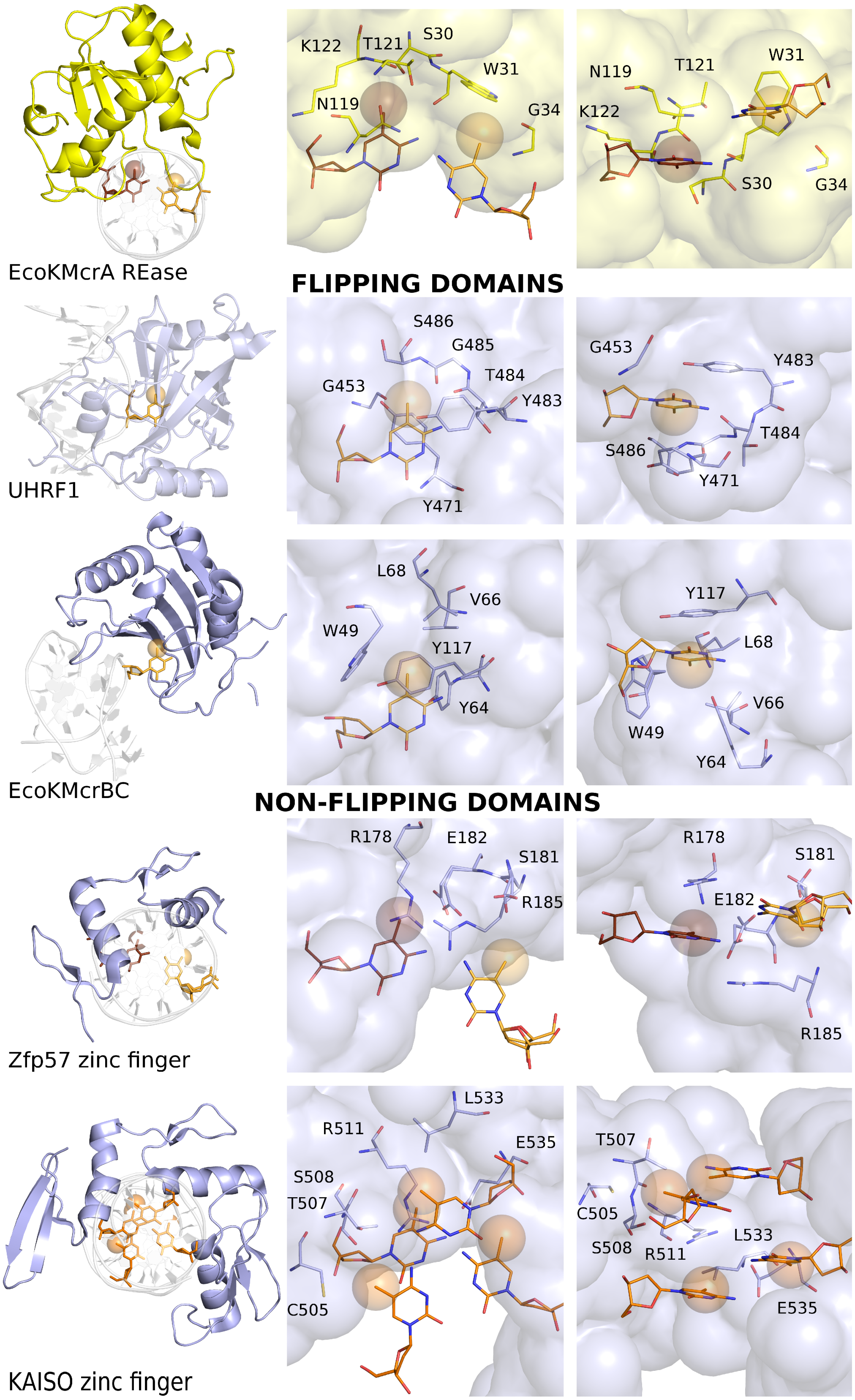
EcoKMcrA REase
K122
T121
S30
W31
N119
G34
K122
S30
FLIPPING DOMAINS
S486
G485
G453
T484
Y483
Y471
T484
UHRF1
L68
V66
W49
Y117
Y64
EcoKMcrBC
NON-FLIPPING DOMAINS
R178
E182
S181
R185
Zfp57 zinc finger
L533
R511
E535
S508
T507
C505
KAISO zinc finger

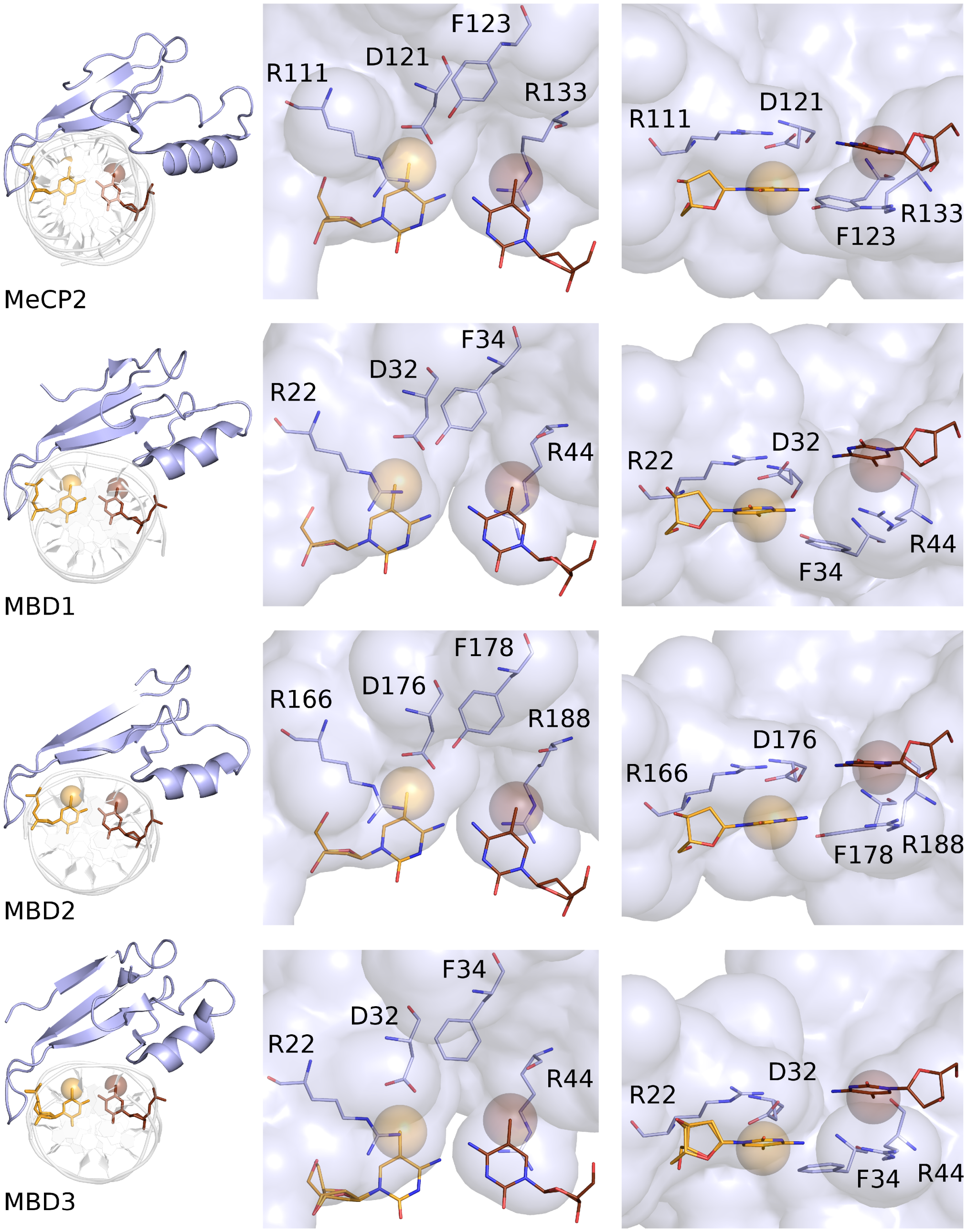

Fig. S13: Methyl group binding by protein domains. (A) Methyl group recognition by EcoKMcrA (this work), in the context of the flipped out base by the UHRF1 SRA domain (7-9), and EcoKMcrBC N-terminal domain (10), and in the context of double stranded DNA by Zfp57 (11) and KAISO (12) zinc fingers, MeCP2 and MBD proteins (13,14).

Fig. S14

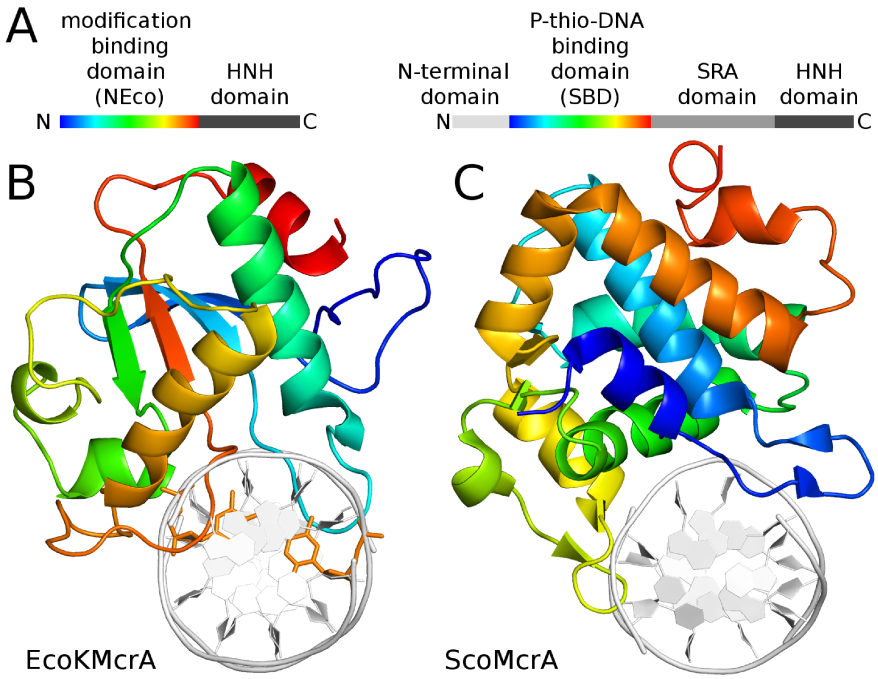

Fig. S14: Comparison of EcoKMcrA and ScoMcrA domain organization and DNA binding mode. **(A)** Schematic view of the two protein sequences. **(B,C)** Overall structure of **(B)** EcoKMcrA N-terminal domain with the central β-sheet and **(C)** predominantly α-helical domain of ScoMcrA selective for phosphorothioated DNA (15). The two domains are rainbow-colored from N- to C-terminus.

Supplementary Tables

Table S1: Data collection and refinement statistics

Data collection statistics	**NEco - C^{5m}CGG**	**NEco - T^{5m}CGA**	**NEco - T^{5hm}CGA**
**Data collection statistics**			
Beamline	BESSY 14.1	DESY P14	DESY P14
Space group	P6(1)22	P6(1)22	P6(1)22
Cell dimensions<br>a (Å)<br>c (Å)	 112.4<br>155.5	 112.9<br>155.8	 113.4<br>156.37
Wavelength (Å)	0.9184	0.9793	0.9195
Resolution range (Å)<br>Lowest shell<br>Highest shell	46 - 2.64<br>46 - 7.83<br>2.80 - 2.64	33.4 - 2.07<br>33.4 – 6.11<br>2.19 – 2.07	33.5 - 2.21<br>33.5 - 6.51<br>2.34 – 2.21
Total reflections	283 344	1 409 324	1 085 536
Unique reflections	17 671	36 261	30 298
Completeness (%)*	99.8 (99.0, 99.2)	99.5 (98.7, 98.6)	99.2 (98.5, 95.4)
Multiplicity *	16.0 (14.0, 16.7)	38.9 (35.0, 38.9)	35.8 (33.3, 20.8)
Mean $I/\sigma I$ *	12.5 (41.1, 1.97)	30.2 (110.0, 1.98)	38.2 (133.0, 1.91)
R (%)*	22.3 (5.4, 146.3)	10.3 (3.2, 229.9)	8.4 (2.6, 176.1)
R(meas) (%)*	23.0 (5.6, 150.9)	10.5 (3.3, 232.9)	8.6 (2.6, 180.2)
$CC_{1/2}$ (%)*	99.7 (99.8, 76.4)	100.0 (100.0, 75.8)	100.0 (100.0, 73.4)
Solvent content (%)	59.4	59.8	60.3
B(iso) from Wilson (Å^2)	50.9	50.3	55.6
**Refinement statistics**			
Protein atoms excluding H #	2320	2484	2425
DNA atoms excluding H #	812	812	816
Solvent molecules	146	431	405
R_{cryst} (%)	19.05	15.90	15.71
R_{free} (%) $	22.92	20.13	20.38
RMSD bond lengths (Å)	0.006	0.006	0.005
RMSD angles (°)	1.05	1.15	1.22
Ramachandran favored region (%)	100.0	100.0	100.0
Ramachandran allowed region (%)	97.6	97.6	97.6
Molprobity clashscore	0.73	1.5	1.9
**PDB code**	**6R64**	**6T21**	**6T22**

* Lowest and highest shell in brackets

\# Double conformations counted separately

$ 5% of reflections were set aside randomly

Table S2: Oligonucleotides used in this study.

Name*	Sequence**	Comment
C^{5m}CGG	5′-AGACCCACGCTCAC$^{\mathbf{5m}}$**C**GGTTCCAGATTTATC-3′<br>3′-TCTGGGTGCGAGTGG$^{\mathbf{5m}}$**C**CAAGGTCTAAATAG-5′	Fully-methylated DNA, optimal sequence context C^{5m}CGG
C^{5hm}CGG	5′-AGACCCACGCTCAC$^{\mathbf{5hm}}$**C**GGTTCCAGATTTATC-3′<br>3′-TCTGGGTGCGAGTGG$^{\mathbf{5hm}}$**C**CAAGGTCTAAATAG-5′	Fully-hydroxymethylated DNA
C^{5hm}CGG/C^{5m}CGG	5′-AGACCCACGCTCAC$^{\mathbf{5hm}}$**C**GGTTCCAGATTTATC-3′<br>3′-TCTGGGTGCGAGTGG$^{\mathbf{5m}}$**C**CAAGGTCTAAATAG-5′	As C^{5m}CGG, but top strand hydroxymethylated
CTGG/C^{5m}CGG	5′-AGACCCACGCTCAC**T** GGTTCCAGATTTATC-3′<br>3′-TCTGGGTGCGAGTG**G**$^{\mathbf{5m}}$**C**CAAGGTCTAAATAG-5′	As C^{5m}CGG, but top stand 5mC replaced by T, forming a T:G mismatch
CTGG/C^{5m}CGG	5′-AGACCCACGCTCAC**T** GGTTCCAGATTTATC-3′<br>3′-TCTGGGTGCGAGTG**A**$^{\mathbf{5m}}$**C**CAAGGTCTAAATAG-5′	As C^{5m}CGG, but one ^{5m}C:G base pair replaced by a T:A base pair
C^{5m}CGG/CCGG	5′-AGACCCACGCTCAC$^{\mathbf{5m}}$**C**GGTTCCAGATTTATC-3′<br>3′-TCTGGGTGCGAGTGGCCAAGGTCTAAATAG-5′	Hemi-methylated CCGG DNA
CCGG	5′-AGACCCACGCTCACCGGTTCCAGATTTATC-3′<br>3′-TCTGGGTGCGAGTGGCCAAGGTCTAAATAG-5′	Non-methylated CCGG DNA
G^{5m}CGG/C^{5m}CGC	5′-AGACCCACGCTCAG$^{\mathbf{5m}}$**C**GGTTCCAGATTTATC-3′<br>3′-TCTGGGTGCGAGTCG$^{\mathbf{5m}}$**C**CAAGGTCTAAATAG-5′	Fully-methylated DNA, suboptimal sequence context G^{5m}CGG/C^{5m}CGC
G^{5m}CGC	5′-AGACCCACGCTCAG$^{\mathbf{5m}}$**C**GCTTCCAGATTTATC-3′<br>3′-TCTGGGTGCGAGTCG$^{\mathbf{5m}}$**C**GAAGGTCTAAATAG-5′	Fully-methylated DNA, suboptimal sequence context G^{5m}CGC
T^{5m}CGA	5′-AGACCCACGCTCAT$^{\mathbf{5m}}$**C**GATTCCAGATTTATC-3′<br>3′-TCTGGGTGCGAGTAG$^{\mathbf{5m}}$**C**TAAGGTCTAAATAG-5′	Fully-methylated DNA, optimal sequence context T^{5m}CGA
C^{5m}CGA/T^{5m}CGG	5′-AGACCCACGCTCAC$^{\mathbf{5m}}$**C**GATTCCAGATTTATC-3′<br>3′-TCTGGGTGCGAGTGG$^{\mathbf{5m}}$**C**TAAGGTCTAAATAG-5′	Fully-methylated DNA, optimal sequence context C^{5m}CGA/T^{5m}CGG

* Symmetric complementary sequences are omitted.
** Nucleotides forming the 5′-Y^{5m}CGR-3′ recognition sequence and its variants are underlined.

Table S3: Similarity of various EcoKMcrA structures (rmsd in Å). For statically disordered residues only the A conformation has been used in the rmsd assessment.

A) protein

		NEco in full length EcoKMcrA		NEco in complex with $C^{5m}CGG$		NEco in complex with $T^{5m}CGA$		NEco in complex with $T^{5hm}CGA$	
		mol A	mol B	mol A	mol B	mol A	mol B	mol A	mol B
NEco in full length EcoKMcrA	mol A	-							
	mol B	1.47	-						
NEco in complex with $C^{5m}CGG$	mol A	1.51	1.31	-					
	mol B	1.17	1.27	0.81	-				
NEco in complex with $T^{5m}CGA$	mol A	1.54	1.38	0.40	0.87	-			
	mol B	1.21	1.36	0.85	0.64	0.87	-		
NEco in complex with $T^{5hm}CGA$	mol A	1.55	1.33	0.43	0.84	0.40	0.84	-	
	mol B	1.22	1.33	0.85	0.50	0.91	0.57	0.86	-

B) DNA

		NEco in complex with $C^{5m}CGG$		NEco in complex with $T^{5m}CGA$		NEco in complex with $T^{5hm}CGA$	
		mol A	mol B	mol A	mol B	mol A	mol B
NEco in complex with $C^{5m}CGG$	mol A	-					
	mol B	0.80	-				
NEco in complex with $T^{5m}CGA$	mol A	0.48	0.85	-			
	mol B	0.91	0.51	0.76	-		
NEco in complex with $T^{5hm}CGA$	mol A	0.49	0.86	0.11	0.76	-	
	mol B	0.91	0.53	0.76	0.14	0.76	-

C) protein and DNA

		NEco in complex with $C^{5m}CGG$		NEco in complex with $T^{5m}CGA$		NEco in complex with $T^{5hm}CGA$	
		mol A	mol B	mol A	mol B	mol A	mol B
NEco in complex with $C^{5m}CGG$	mol A	-					
	mol B	0.84	-				
NEco in complex with $T^{5m}CGA$	mol A	0.43	0.89	-			
	mol B	0.91	0.62	0.87	-		
NEco in complex with $T^{5hm}CGA$	mol A	0.45	0.87	0.35	0.86	-	
	mol B	0.92	0.52	0.91	0.49	0.88	-

Table S4: Selected conformational parameters of the DNA oligoduplexes bound to N-terminal domain of EcoKMcrA (NEco). The average, minimal and maximal values of the 6 oligoduplexes observed in the crystal structures are indicated (two NEco-DNA molecules were present in the asymmetric unit of each crystal). Figures in the tables were adopted from the 3DNA manual (12).

A: Local base pair parameters

	**Propeller**			**Buckle**		
**A-DNA**	**-10.5**			**0.0**		
**B-DNA**	**-15.1**			**0.0**		
RNA	-2.08			0.0		
bp	average	min	max	average	min	max
A : T	-6.4	-10.1	-17.7	0.1	-5.4	4.9
C Y : R	**-23.8**	**-22.8**	**-24.0**	**-9.0**	**-16.6**	**-4.4**
$^{5(h)m}$C : G	**13.3**	**10.9**	**8.2**	**-3.0**	**-5.9**	**-1.2**
G : $^{5(h)m}$C	**-25.7**	**-23.0**	**-25.7**	**2.6**	**0.9**	**4.0**
G R : Y	**-19.7**	**-17.8**	**-19.7**	**3.0**	**-4.0**	**15.7**
T : A	-15.0	-17.6	-22.9	2.3	0.3	6.0
	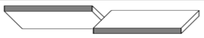			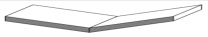		

B: Local base pair step parameters

	**Shift**			**Tilt**		
**A-DNA**	**-0.01**			**0.04**		
**B-DNA**	**0.00**			**0.00**		
RNA	-0.08			-0.44		
bp step	average	min	max	average	min	max
AY / RT	-0.5	-0.7	-0.4	-2.7	-5.1	-1.3
Y$^{5(h)m}$C / GR	**1.5**	**1.2**	**1.7**	**5.5**	**2.4**	**10.1**
$^{5(h)m}$CG / $^{5(h)m}$CG	**-1.2**	**-1.3**	**-1.0**	**-1.2**	**-3.4**	**0.8**
GR / Y$^{5(h)m}$C	**0.5**	**0.3**	**0.7**	**-0.6**	**-3.2**	**1.0**
RT / AY	-0.3	-0.6	-0.1	-0.3	-1.3	2.4
	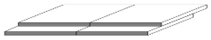			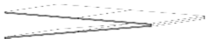		

C: Base pair position and orientation with respect to the helix axis.

	**X-disp**			**Y-disp**			**Incl**		
**A-DNA**	**-4.5**			**0.02**			**22.7**		
**B-DNA**	**0.5**			**0.01**			**2.8**		
RNA	-4.1			0.07			15.5		
bp step	ave	min	max	ave	min	max	ave	min	max
AC/GT	0.1	-0.1	0.5	0.4	0.0	0.9	-9.9	-12.0	-8.3
**Y$^{5(h)m}$C/GR**	**1.2**	**0.7**	**1.6**	**-1.5**	**-2.1**	**-0.4**	**1.6**	**0.4**	**2.4**
**$^{5(h)m}$CG/$^{5(h)m}$CG**	**2.0**	**1.8**	**2.4**	**1.8**	**1.4**	**2.2**	**-8.6**	**-9.7**	**-6.2**
**GR/Y$^{5(h)m}$C**	**0.2**	**-0.2**	**0.9**	**-0.8**	**-1.4**	**-0.3**	**-3.2**	**-7.6**	**-0.1**
GT/AC	-0.1	-0.3	0.1	0.4	-0.1	1.2	-4.5	-6.8	-2.6
	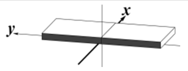			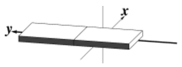			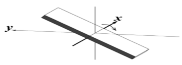		

Table S5: Dissociation constants for the DNA complexes of the studied NEco variants determined by EMSA.

NEco variant	K_D (nM) *		
	Fully-methylated DNA ($C^{5m}CGG$)	Fully-hydroxymethylated DNA ($C^{5hm}CGG$)	Hemi-methylated DNA ($C^{5m}CGG/CCGG$)
wt	18 ± 3	80 ± 20	n. d.
S30L	4000 ± 300	n. d.	n. d.
S30V	2100 ± 400	n. d.	n. d.
S30A	60 ± 20	n. d.	n. d.
W31A	34 ± 6	n. d.	130 ± 40
W31S	43 ± 6	n. d.	130 ± 30
W31H	40 ± 6	n. d.	n. d.
W31F	120 ± 40	n. d.	n. d.
W31Y	47 ± 7	n. d.	n. d.
W31I	1000 ± 200	n. d.	n. d.
W31L	n. d. **	n. d.	n. d.
W31V	n. d.	n. d.	n. d.
N119A	750 ± 100	n. d.	n. d.

* EMSA experiments were performed under standard conditions (3 or more independent experiments, 10 nM DNA and variable protein concentrations). K_D values for each experiment were calculated as described in Supplementary Methods. Reported values are average K_D values ± 1 SE.

** n. d. – K_D below the detection limit. No quantifiable complex formed, K_D not determined.

References:

1. Czapinska, H., Kowalska, M., Zagorskaitė, E., Manakova, E., Slyvka, A., Xu, S.-y., Siksnys, V., Sasnauskas, G. and Bochtler, M. (2018) Activity and structure of EcoKMcrA. *Nucleic Acids Research*, gky731-gky731.
2. Sasnauskas, G., Kauneckaite, K. and Siksnys, V. (2018) Structural basis of DNA target recognition by the B3 domain of Arabidopsis epigenome reader VAL1. *Nucleic Acids Res*, **46**, 4316-4324.
3. Sokolowska, M., Czapinska, H. and Bochtler, M. (2009) Crystal structure of the beta beta alpha-Me type II restriction endonuclease Hpy99I with target DNA. *Nucleic Acids Res*, **37**, 3799-3810.
4. Cuypers, M.G., Robertson, R.M., Knipling, L., Waddell, M.B., Moon, K., Hinton, D.M. and White, S.W. (2018) The phage T4 MotA transcription factor contains a novel DNA binding motif that specifically recognizes modified DNA. *Nucleic Acids Res*, **46**, 5308-5318.
5. Chevalier, B.S., Kortemme, T., Chadsey, M.S., Baker, D., Monnat, R.J. and Stoddard, B.L. (2002) Design, activity, and structure of a highly specific artificial endonuclease. *Mol Cell*, **10**, 895-905.
6. Anton, B.P. and Raleigh, E.A. (2004) Transposon-mediated linker insertion scanning mutagenesis of the Escherichia coli McrA endonuclease. *J Bacteriol*, **186**, 5699-5707.
7. Avvakumov, G.V., Walker, J.R., Xue, S., Li, Y., Duan, S., Bronner, C., Arrowsmith, C.H. and Dhe-Paganon, S. (2008) Structural basis for recognition of hemi-methylated DNA by the SRA domain of human UHRF1. *Nature*, **455**, 822-825.
8. Han, T., Yamada-Mabuchi, M., Zhao, G., Li, L., Liu, G., Ou, H.Y., Deng, Z., Zheng, Y. and He, X. (2015) Recognition and cleavage of 5-methylcytosine DNA by bacterial SRA-HNH proteins. *Nucleic Acids Res*, **43**, 1147-1159.
9. Hashimoto, H., Horton, J.R., Zhang, X., Bostick, M., Jacobsen, S.E. and Cheng, X. (2008) The SRA domain of UHRF1 flips 5-methylcytosine out of the DNA helix. *Nature*, **455**, 826-829.
10. Sukackaite, R., Grazulis, S., Tamulaitis, G. and Siksnys, V. (2012) The recognition domain of the methyl-specific endonuclease McrBC flips out 5-methylcytosine. *Nucleic Acids Res*, **40**, 7552-7562.
11. Liu, Y., Toh, H., Sasaki, H., Zhang, X. and Cheng, X. (2012) An atomic model of Zfp57 recognition of CpG methylation within a specific DNA sequence. *Genes Dev*, **26**, 2374-2379.
12. Buck-Koehntop, B.A., Stanfield, R.L., Ekiert, D.C., Martinez-Yamout, M.A., Dyson, H.J., Wilson, I.A. and Wright, P.E. (2012) Molecular basis for recognition of methylated and specific DNA sequences by the zinc finger protein Kaiso. *Proc Natl Acad Sci U S A*, **109**, 15229-15234.
13. Ho, K.L., McNae, I.W., Schmiedeberg, L., Klose, R.J., Bird, A.P. and Walkinshaw, M.D. (2008) MeCP2 binding to DNA depends upon hydration at methyl-CpG. *Mol Cell*, **29**, 525-531.
14. Liu, K., Xu, C., Lei, M., Yang, A., Loppnau, P., Hughes, T.R. and Min, J. (2018) Structural basis for the ability of MBD domains to bind methyl-CG and TG sites in DNA. *J Biol Chem*, **293**, 7344-7354.
15. Liu, G., Fu, W., Zhang, Z., He, Y., Yu, H., Wang, Y., Wang, X., Zhao, Y.L., Deng, Z., Wu, G. *et al.* (2018) Structural basis for the recognition of sulfur in phosphorothioated DNA. *Nat Commun*, **9**, 4689.
